# Identification of QTL markers contributing to plant growth, oil yield and fatty acid composition in the oilseed crop *Jatropha curcas* L.

**DOI:** 10.1186/s13068-015-0326-8

**Published:** 2015-09-25

**Authors:** Andrew J. King, Luis R. Montes, Jasper G. Clarke, Jose Itzep, Cesar A. A. Perez, Raymond E. E. Jongschaap, Richard G. F. Visser, Eibertus N. van Loo, Ian A. Graham

**Affiliations:** Department of Biology, Centre for Novel Agricultural Products, University of York, York, YO10 5DD UK; Biocombustibles de Guatemala, Guatemala Ciudad, Guatemala; Wageningen UR Plant Breeding, Wageningen University and Research Centre, PO Box 386, 6700 AJ Wageningen, The Netherlands; Graduate School of Experimental Plant Sciences, Wageningen University, Wageningen, The Netherlands; Wageningen UR Agrosystems Research, Wageningen University and Research Centre, PO Box 16, 6708 AP Wageningen, The Netherlands; Facultad de Agronomia, Universidad de San Carlos de Guatemala, Edifico T-8 y T-9 Ciudad Universitaria zona 12, Guatemala Cuidad, Guatemala

**Keywords:** *Jatropha curcas*, Linkage mapping, QTL analysis, Oil content, Seed weight, Seed yield

## Abstract

**Background:**

Economical cultivation of the oilseed crop *Jatropha curcas* is currently hampered in part due to the non-availability of purpose-bred cultivars. Although genetic maps and genome sequence data exist for this crop, marker-assisted breeding has not yet been implemented due to a lack of available marker–trait association studies. To identify the location of beneficial alleles for use in plant breeding, we performed quantitative trait loci (QTL) analysis for a number of agronomic traits in two biparental mapping populations.

**Results:**

The mapping populations segregated for a range of traits contributing to oil yield, including plant height, stem diameter, number of branches, total seeds per plant, 100-seed weight, seed oil content and fatty acid composition. QTL were detected for each of these traits and often over multiple years, with some variation in the phenotypic variance explained between different years. In one of the mapping populations where we recorded vegetative traits, we also observed co-localization of QTL for stem diameter and plant height, which were both overdominant, suggesting a possible locus conferring a pleotropic heterosis effect. By using a candidate gene approach and integrating physical mapping data from a recent high-quality release of the *Jatropha* genome, we were also able to position a large number of genes involved in the biosynthesis of storage lipids onto the genetic map. By comparing the position of these genes with QTL, we were able to detect a number of genes potentially underlying seed traits, including phosphatidate phosphatase genes.

**Conclusions:**

The QTL we have identified will serve as a useful starting point in the creation of new varieties of *J. curcas* with improved agronomic performance for seed and oil productivity. Our ability to physically map a significant proportion of the *Jatropha* genome sequence onto our genetic map could also prove useful in identifying the genes underlying particular traits, allowing more controlled and precise introgression of desirable alleles and permitting the pyramiding or stacking of multiple QTL.

**Electronic supplementary material:**

The online version of this article (doi:10.1186/s13068-015-0326-8) contains supplementary material, which is available to authorized users.

## Background

*Jatropha curcas* L. is a perennial oilseed crop which is suitable for cultivation in tropical and sub-tropical regions [1]. At present, the economic cultivation of this orphan crop is hampered by a number of factors. As *J. curcas* cultivation has only occurred sporadically on a relatively small scale, there is currently limited knowledge of the agronomy of this crop, and the reported yields obtained so far vary significantly. While seed yields of up to 3–4 tonnes per hectare can be achieved under controlled conditions [2–4], “farm” yields are typically much lower [5, 6] and well below “projections” that have been indicated in a number of reports (summarized in Heller [7]). Economic cultivation of *Jatropha* has also been hampered by the lack of purpose-bred cultivars and the reliance on genetically homogeneous plants that are likely to be descended from very limited germplasm that was originally transported to Cape Verde by the Portuguese during colonial times [7]. *J. curcas* is native to Mesoamerica, and analyses performed using robust markers such as amplified fragment length polymorphism (AFLP), single nucleotide polymorphisms (SNP) and simple sequence repeats (SSR) have indicated that the material currently grown in Africa, Asia and South America is almost clonal [9–11]. Significant genetic variation, however, has been reported in Mesoamerica, particularly in Guatemala and the state of Chiapas in Mexico [9, 10, 12, 13]. These Mesoamerican provenances of *J. curcas* therefore represent a valuable germplasm resource for the purpose of breeding. As a first step in developing a molecular breeding programme for the improvement of *J. curcas*, we recently constructed a genetic linkage map for this species [14]. We have previously used this map to identify, to within 2.3 cM, a locus responsible for the loss of phorbol ester biosynthesis in “non-toxic” types of *J. curcas*. These phorbol esters are not removed by conventional seed meal processing methods and make the use of the protein-rich seed meal obtained from most “varieties” of *J. curcas* unsuitable for use as animal feed [9, 15]. As well as identifying loci controlling qualitative Mendelian traits, mapping populations can also be used to find quantitative trait loci (QTL), i.e. regions of the genome contributing to complex multigenic traits which are scored as continuous data. QTL mapping has previously been conducted on an interspecific cross between *J. curcas* and *J. integerrima*, resulting in the identification of loci contributing to seed weight, fatty acid composition and vegetative growth characteristics (including height and branching) [16, 17]. Although these QTL are useful for identifying beneficial (as well as non-desirable) loci for breeding of new plant varieties containing chromosomal introgressions from *J. integerrima*, this interspecific mapping population approach cannot identify beneficial alleles present within the *J. curcas* germplasm. For this purpose, we collected phenotypic data from two different mapping populations incorporating “wild” provenances collected from Guatemala. Within these populations we identified QTL for a number of agronomic traits including plant height, stem diameter, canopy area, number of branches, 100-seed weight and seed oil content, many of which appeared to be stable over multiple harvest years. Pyramiding of these QTL in other genetic backgrounds could lead to the creation of improved cultivars more suited to the commercial production of vegetable oil and animal feed from this orphan crop. We also present an updated genetic linkage map for *Jatropha* containing additional markers, onto which we mapped scaffolds from a recent high-quality draft of the *J. curcas* genome [18], and discuss the utility of this approach in identifying candidate genes underlying important QTL.

## Results and discussion

### An updated genetic linkage map for *Jatropha curcas*

**Fig. 1:**
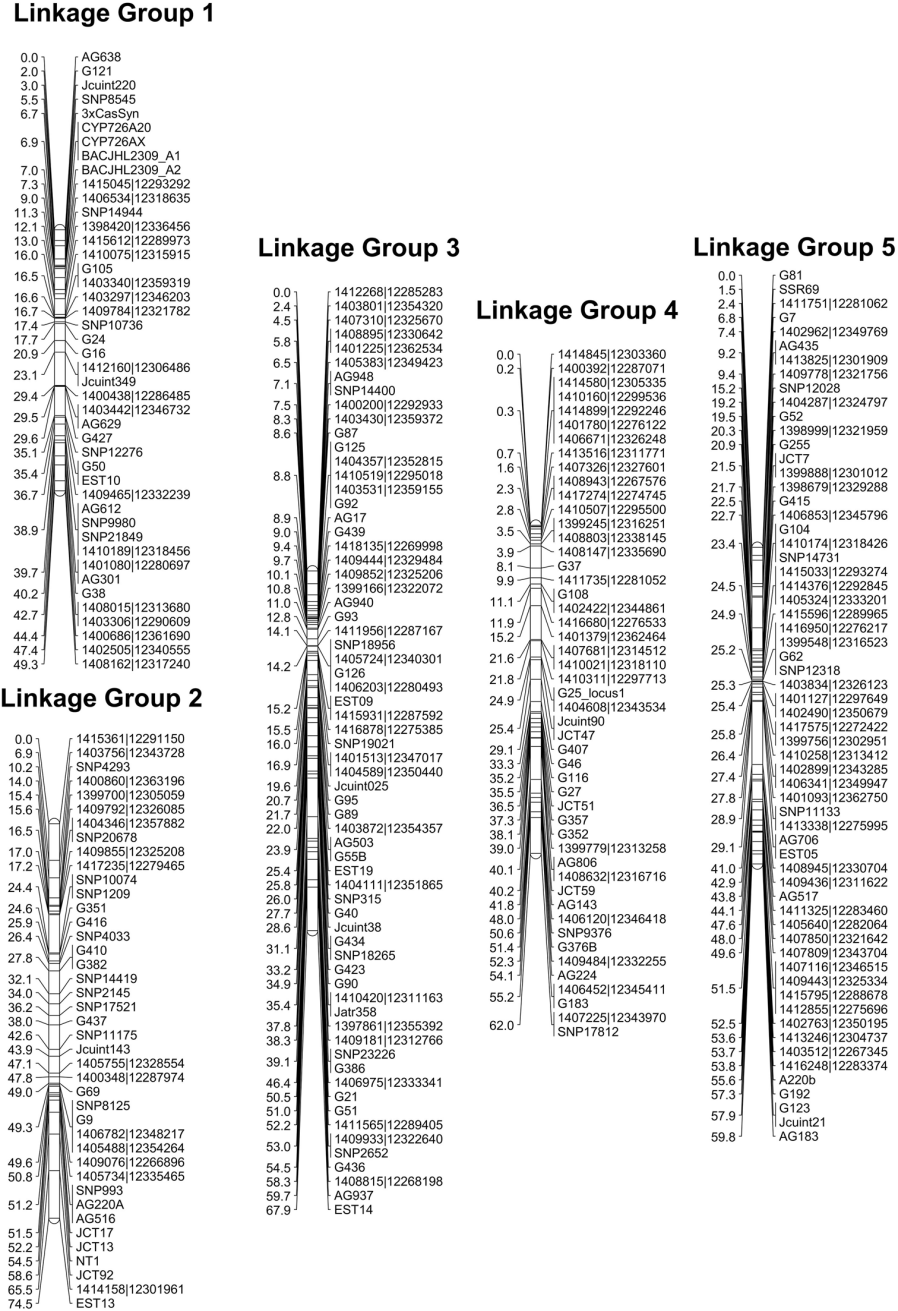
Linkage groups 1–5 of the combined *J. curcas* linkage map. Positions of markers are shown in cM (Kosambi)

**Fig. 2:**
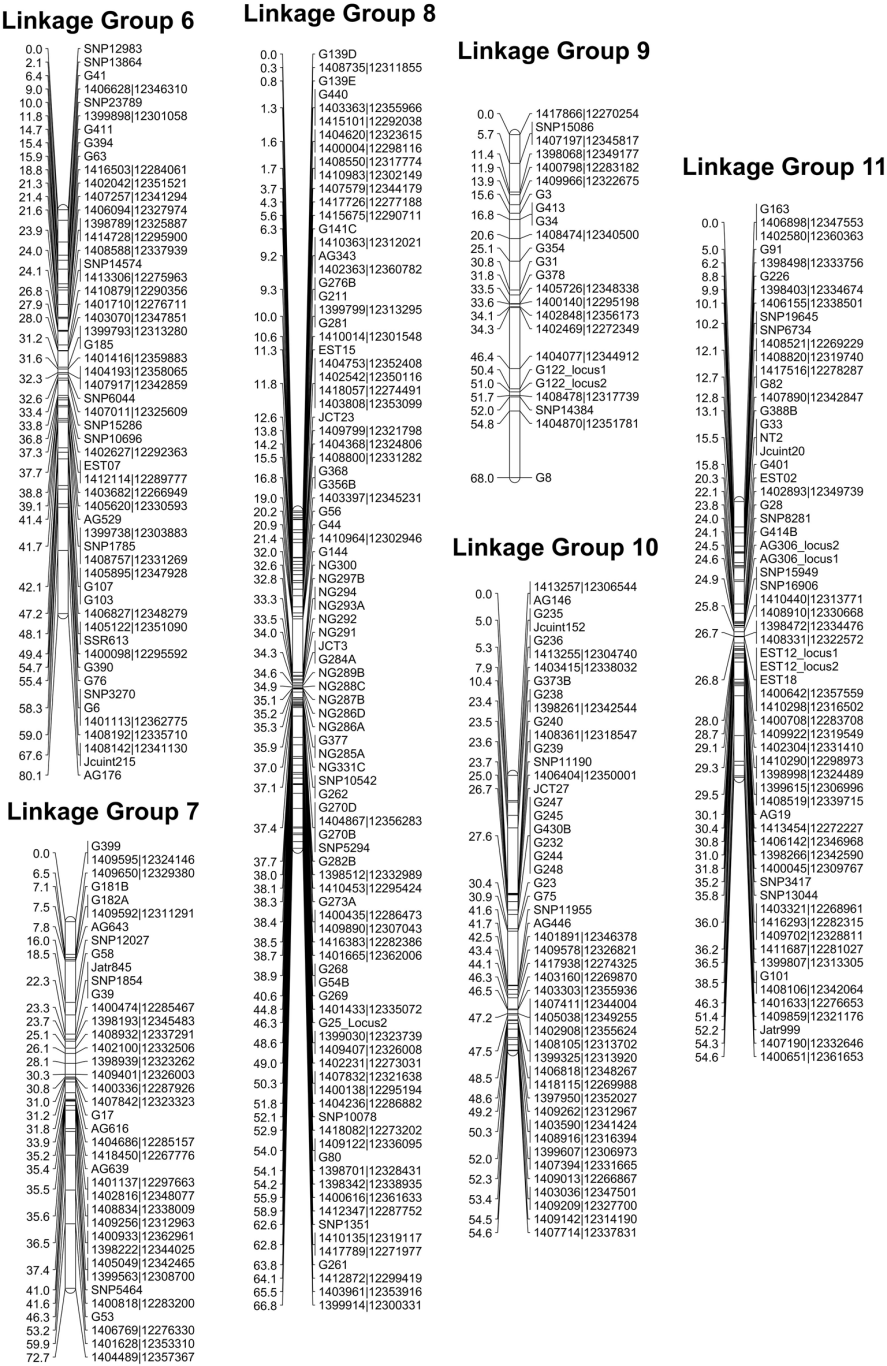
Linkage groups 6–11 of the combined *J. curcas* linkage map. Positions of markers are shown in cM (Kosambi)

We recently published the first intraspecies linkage map for *J. curcas* [14]. The combined map, which was based on four F_2_ mapping populations, contained 502 markers spanning a total distance of 717 cM. To improve the density of individual maps and add candidate genes that may contribute to specific traits, we developed a number of additional SSR markers which are detailed in Additional file 1: Table S1. The revised genetic linkage map, which now contains 587 markers spanning a total distance of 673 cM, is shown in Figs. 1 and 2. A summary of the markers, marker densities and genetic distances for each of the linkage groups is shown in Table 1. The increase in the number of markers, together with a small reduction in the overall calculated map length, has resulted in a modest improvement in mean marker density of 0.3 cM; our latest map has a density of 1.2 cM per marker or 1.5 cM per unique locus, compared with 1.5 and 1.8 cM, respectively, in our previous map.

**Table 1:** Summary statistics of the *J. curcas* combined linkage map

Linkage group	Markers	Unique loci	Length (cM)	Marker density (All)	Marker density (Unique)	Genome mapped (Mbp)	Gene models mapped
1	44	35	49.3	1.1	1.4	12.3	1495
2	41	34	74.5	1.9	2.3	15.8	1609
3	66	52	67.9	1.0	1.3	20.4	1435
4	49	37	62.0	1.3	1.7	11.7	1343
5	62	47	59.8	1.0	1.3	14.3	1661
6	55	55	43.0	0.8	0.8	15.9	1960
7	39	32	72.7	1.9	2.3	19.1	2007
8	94	71	66.8	0.7	1.0	13.5	1737
9	24	23	68.0	3.0	3.1	9.9	1242
10	49	32	54.6	1.1	1.8	14.0	1343
11	64	47	54.6	0.9	1.2	15.2	1620
Total	587	465	673.2	1.2	1.5	162.2	17,452

Previously, using the draft genome assembly released by the Kazusa DNA Research Institute [19, 20], we were able to physically map 17 Mbp (of 297 Mbp) of genome sequence against our genetic linkage map. Within this 17 Mbp were 3077 of the 39,277 predicted gene models [14]. This represents 5.7 % of the genome and 7.8 % of the predicted genes for this version of genome assembly. The ability to map a greater proportion of the genome would be beneficial in allowing the position of candidate genes likely to correspond to particular traits to be mapped. Recently, the Chinese Academy of Sciences (CAS) has also released a *J. curcas* genome [18]. This genome was obtained from sequencing to a depth of 189-fold, and contains scaffolds with an N50 of 746,835 compared to the Kazusa DNA Research Institute version 4.5, which has an N50 of 15,950. This improved genome assembly provided us with the opportunity to physically map a substantial amount of the genome against our genetic linkage map. After conducting BlastN searches of our molecular markers against this new version of the genome, we were able to map a total of 162 Mbp of the predicted 318 Mbp (i.e. 51 %) of the CAS *Jatropha* genome assembly (Table 2 and Additional file 2: Tables S2–S13). This is similar to the value obtained by Wu et al. using our previous generation of the map [18]. In a few instances we observed that some scaffolds mapped to more than one linkage group. This may be due to misassemblies in the published genome sequence or segmental chromosome duplications. In general, however, our mapping order was highly consistent with this draft genome sequence. The scaffolds that we were able to map contained 17,452 of 27,172 predicted protein encoding sequences (64 %) contained within the CAS *Jatropha* genome (Table 1 and Additional file 2: Table S2).

**Table 2:**
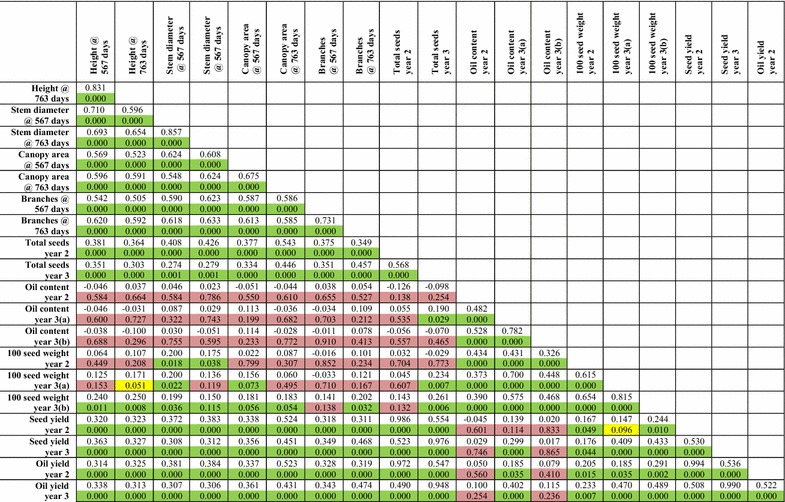
Pearson correlations and *p* values for vegetative and oil yield traits in mapping population G51 × CV

The upper uncoloured cells contain the *R* values. The lower coloured cells contain the *p* values. Cells shaded in green represent correlations with a *p* value <0.05, cells shaded in yellow represent a *p* value of between 0.05 and 0.10, whereas cells shaded in red represent a *p* value >0.10 (non-significant). Details of data collection and calculation for each trait are provided in “Methods”

### Positioning markers for storage lipid biosynthesis candidate genes onto the linkage map

**Fig. 3:**
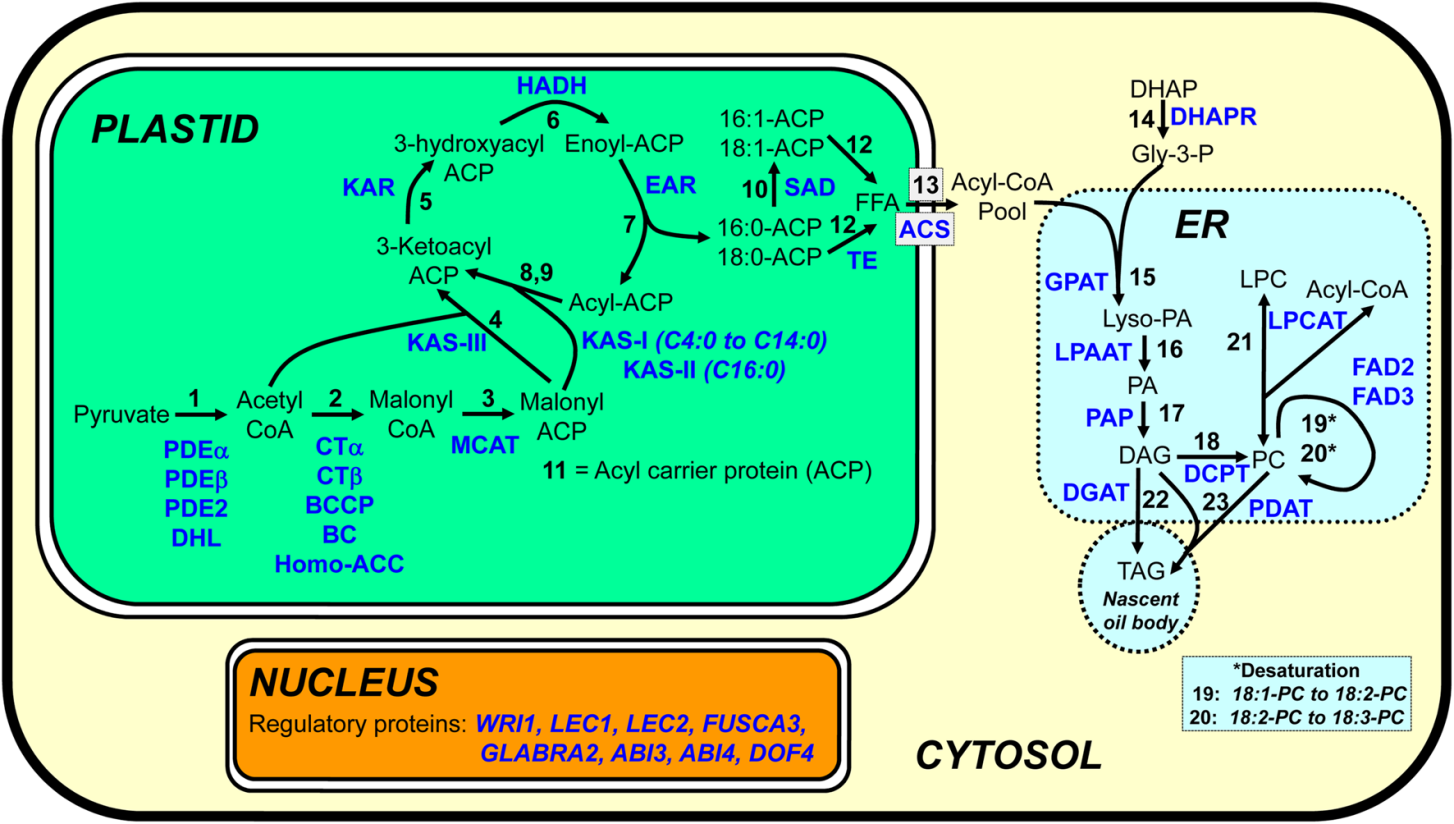
Summary of mapped candidate genes involved in the biosynthesis of storage lipids in *J. curcas*. The genes, indicated in *blue text*, are as follows: *Plastid*—(*1*) *PDEα* α-subunit of the pyruvate dehydrogenase (PDH) complex, *PDEβ* β-subunit of the PDH complex, *PDE2* dihydrolipoyl transacetylase component of the PDH complex and *PDE3* dihydrolipoamide dehydrogenase component of the PDH complex; (*2*) *CTα* α-subunit of the heteromeric acetyl-coA carboxylase (ACCase) complex, *CTβ* β-subunit of the heteromeric ACCase complex, *BCCP* biotin carboxyl carrier protein and *BC* biotin-carboxylase subunit of the heteromeric ACCase complex; (*3*) *MCAT* malonyl-CoA:ACP malonyltransferase (*4*,*8* and *9*) and *KAS* 3-ketoacyl-ACP synthase; (*5*) *KAR* 3-ketoacyl-ACP reductase; (*6*) *HADH* 3-hydroxylacyl-ACP dehydratase; (*7*) *EAR* enoyl-ACP reductase; (*10*) *SAR* stearoyl-ACP reductase; (*11*) *ACP* acyl carrier protein; (*12*) *ACP-TE* acyl-ACP thioesterase; (*13*) *ACS* acyl-CoA synthetase. *Cytosol* **—**(*14*) *DHAPR* dihydroxyacetone phosphate reductase. Endoplasmic Reticulum (*ER*)**—**(*15*) *GPAT* glycerol-3-phosphate acyltransferase; (*16*) *LPAAT* lysophosphatidic acid acyltransferase; (*17*) *PAP* phosphatidate phosphatase; (*18*) *DCPT* diacylglycerol:choline phosphatidyltransferase; (*19*, *20*) *FAD* fatty acid desaturase; (*21*) *LPCAT* 1-acylglycerol-3-phosphocholine acyltransferase; (*22*) *DGAT* diacylglycerol acyltransferase; (*23*) *PDAT* phospholipid:diacylglycerol acyltransferase. *Nucleus* **—**regulatory proteins including Wrinkled1 (*WRI1*), Leafy Cotyledon 1 & 2 (*LEC1* & *LEC2*), *FUSCA3*, *GLABRA2*, Abscisic Acid Insensitive 3 & 4 (*ABI3* & *ABI4*) and *DOF4*. Abbreviations used for pathway intermediates (*black*) include *DHAP* dihydroxyacetone phosphatase, *Gly-3-P* glycerol-3-phosphate, *Lyso-PA* lysophosphatidic acid, *PA* phosphatidic acid, *DAG* diacylglycerol, *TAG* triacylglycerol, *PC* phosphatidylcholine and *LPC* lysophosphatidylcholine

To locate the positions of lipid biosynthesis genes onto our linkage map, we first identified the orthologues of *Arabidopsis* genes known or suspected to be involved in de novo plastidial lipid biosynthesis and the pathway for the conversion of acyl-CoA into triglycerides, the principal storage lipid in seeds. A diagrammatic representation of these pathways is shown in Fig. 3. In addition to enzymes, we included a number of regulatory proteins. The candidate gene list was compiled from the Arabidopsis Acyl-Lipid Metabolism Website [21]. The genes were identified using BlastP searches of the peptide sequence data for *J. curcas* contained on GenBank. In addition to a number of markers that we developed in close proximity to these candidate genes, we also used the combined genetic and physical map shown in Additional file 2, and the genetic or physical map produced for the interspecific crosses [18, 22], and thus were able to identify the positions of almost all of the lipid biosynthesis candidate genes. These genes could potentially be utilized for molecular breeding by the targeted development of additional SNP or SSR markers in the flanking regions of these genes (Additional file 3: Table S14). The limited number of genes involved in lipid biosynthesis that we were unable to map included one isoform of the plasitidial enoyl-acyl carrier protein reductase (step 7 in Fig. 3) which resides on a scaffold we could not map, and a glycerol-3-phosphate acyltransferase isoform and *Wrinkled1* transcription factor isoform which both mapped to part of a (possibly misassembled) scaffold that may be part of linkage group 3 or 8.

### Both vegetative traits and seed weight contribute to the oil yield in mapping population G51 × CV

The F_2_ mapping population G51 × CV, which has one “wild” partially heterozygous parent (G51, heterozygous at 46 % of markers) and a fully homozygous “Cape Verde”-like parent, was created primarily for the identification of seed oil content QTL, based on contrasting phenotypes we observed for the parents of these plants (36.9 % oil in G51, 26.0 % oil in CV). However, we also collected data for various other traits in the field including plant height, stem diameter, canopy area, number of branches and number of seeds produced (see “Methods”). Normal, or near-normal distributions were observed for the majority of these traits (Additional file 4: Figure S1). To determine the relationship between these variables and the final calculated oil yields per plant, Pearson correlation coefficients were calculated (Table 2). For the final calculated oil yields, almost all of the traits produced significant positive correlations. Within the vegetative traits for example, the number of branches at 763 days (*R* = 0.474) and canopy area at 763 days (*R* = 0.431) produced the highest correlations for year 3 calculated oil yields. These correlations were very similar to those observed for total seeds per plant in year 3 (*R* = 0.457 and 0.446), suggesting that the yield correlations are most closely linked to a higher number of seeds produced in plants showing stronger vegetative growth. Unsurprisingly, the total number of seeds produced per plant was the most significant contributor to the final seed yield (*R* = 0.972 and *R* = 0.948 for years 2 and 3), indicating that for mapping population G51 × CV, the number of seeds per plant is more important than the amount of oil per seed. Nonetheless, 100-seed weights also produced significant correlations with the calculated oil yields (*R* = 0.205 to *R* = 0.489), as did seed oil content in the first harvest for year 3 (*R* = 0.402). Interestingly, for the year 3 data, the total number of seeds per plant also produced a weak but positive correlation with 100-seed weights, indicating that the plants producing more seed do not appear to allocate fewer resources to each seed. Similarly, oil content and seed number either had no correlation or a weak positive correlation (*R* = 0.190 for total seeds in year 3 and oil content in year 3, harvest 1), showing producing more seeds does not reduce the amount of oil stored in the seed.

Overall, the data for this mapping population indicate that the final oil yield is a composite trait, and that the vigour of the plants contributes most significantly to oil yield by producing plants with increased number of seeds. However, 100-seed weights and oil content can also make significant contributions to final oil yield. This suggests that there should be significant potential for developing improved varieties of *J. curcas* through the pyramiding of desirable loci.

### Identification of QTL associated with vegetative growth characteristics, in mapping population G51 × CV

**Fig. 4:**
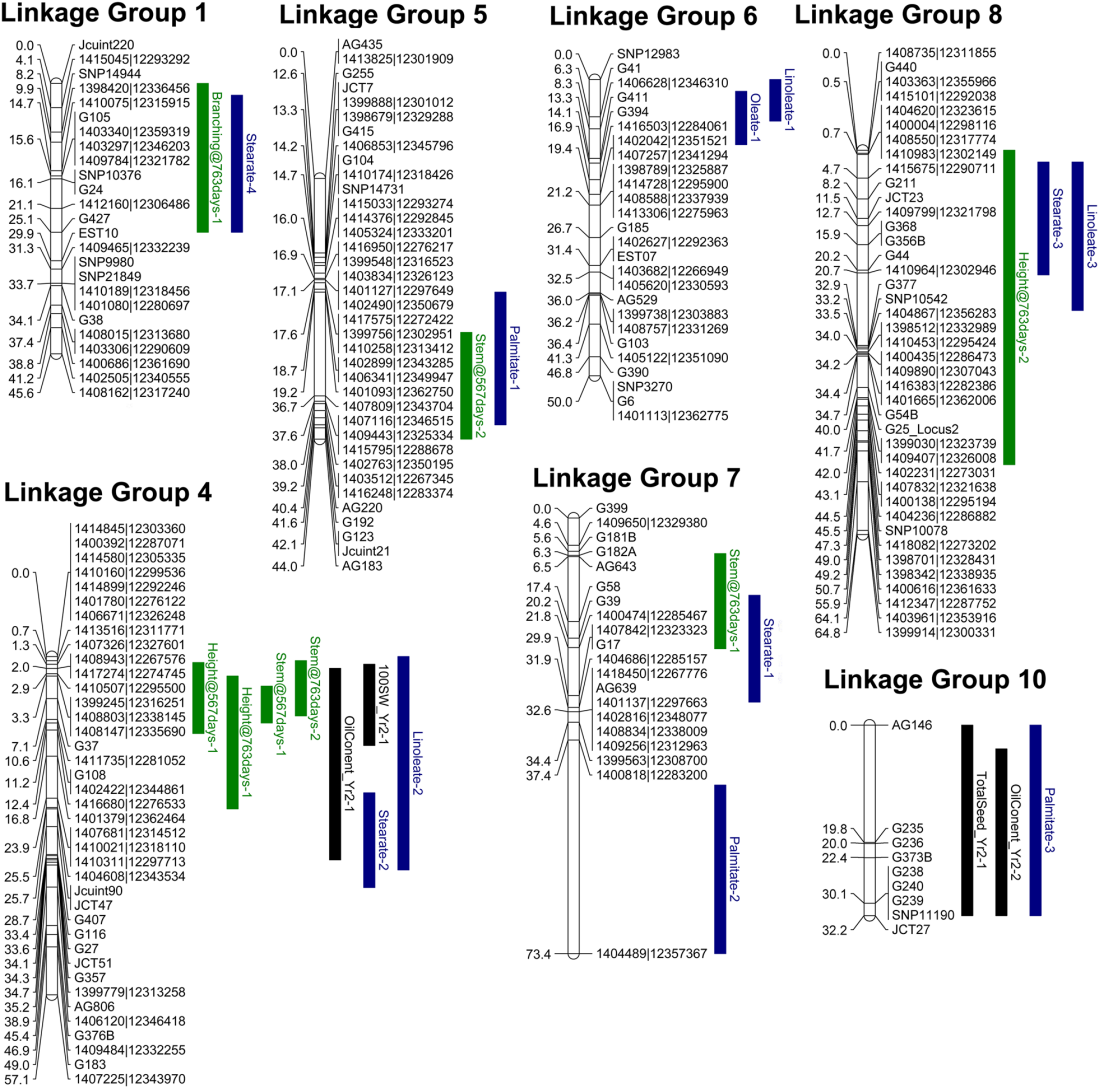
Map of QTL detected in mapping population G51 × CV. QTL shown in *green* relate to vegetative traits (branching, stem diameter and plant height). QTL shown in *black* relate to seed yield traits (seeds per plant, 100-seed weight or oil content). QTL shown in *blue* relate to fatty acid composition in the seed oil (palmitate, stearate, oleate or linoleate). Only linkage groups found to contain QTL are shown

After performing QTL analyses on the data collected from mapping population G51 × CV, we detected a number of QTL underlying vegetative traits (Table 3; Fig. 4; Additional file 5: Figure S2a–e and Additional file 6: Figure S3a–h). QTL for plant height were observed on both linkage group 4 and linkage group 8 (Table 3). The QTL on linkage group 4 was observed at both 567 and 763 days after transplantation from the nursery, accounting for 9.2 and 7.0 % of the phenotypic variance explained (PVE) for these traits, respectively. The height QTL on linkage group 8 was only observed at 763 days, and also accounted for 7.0 % PVE. Both of these QTL were minor and only detected using a significance threshold of *p* = 0.10. The small effects of these height QTL are most likely related to the high level of complexity of this trait. Interestingly, ANOVA analysis of the phenotypes at the height QTL locus on linkage group 4 indicated that this QTL was overdominant, i.e. the heterozygous phenotype was greater than either of the homozygous phenotypes. At the same position of linkage group 4 as the height QTL, we also observed an overdominant QTL corresponding to stem diameter. This accounted for 14.9 and 8.9 % PVE at 567 and 763 days, respectively. A further stem diameter QTL was detected on linkage group 5 at 567 days and linkage group 7 at 763 days. The QTL on linkage group 7 was the largest of these, accounting for 10.2 % PVE. A single dominant QTL for branching was observed on linkage group 1, for which the CV allele had a positive effect. We were unable to detect significant QTL for canopy area, perhaps due to the high level of complexity of the trait. Given the significances of the correlations between the plant vegetative growth traits and the calculated seed and oil yields obtained from the Pearson correlation analysis, the QTL on linkage group 4 for height and stem diameter would be useful targets in a plant breeding programme. The close proximity of these QTL and their similar overdominance indicates that this may be a single locus with a pleotropic effect. However, finer mapping would be required to determine whether these are the same or separate loci. Use of overdominant QTL in plant breeding would require the production of F_1_ hybrid plants for implementation. Due to its monoecious, self-fertile nature, efficient production of F_1_ hybrid seed would require an alternate strategy such as the cytoplasmic male sterility and restorer system [23]. Alternatively, F_1_ plants could be multiplied by vegetative propagation (i.e. from cuttings) or from micropropagation [24].

**Table 3:** Summary of QTL observed for vegetative and oil yield traits in the mapping population G51 × CV

Trait	Observations (n)	Linkage group	Position (cM)	LOD^a^	PVE	Bayes 95 % CI (cM)	Beneficial allele	Effect^b^	QTL plot Additional file 5:	Effect plot Additional file 6:
Height (567 days)	144	4	7.05 (G37)	3.03*	9.2	1.0–13.0	Heterozygous	OD	Fig. S2a	Fig. S3a
Height (763 days)	143	4	8.0	3.19*	7.0	3.34–25.73	Heterozygous	OD	Fig. S2b	Fig. S3b
		8	36.0	3.18*	7.0	0.0–53.0	G51	Dom		Fig. S3c
Stem diameter (567 days)	144	4	7.05 (G37)	4.35***	14.9	5.0–11.21	Heterozygous	OD	Fig. S2c	Fig. S3d
		5	41.1 (G123)	3.23*	8.5	26.0–44.02	CV	Dom		Fig. S3e
Stem diameter (763 days)	143	7	13.0	4.31***	10.2	6.0–22.0	G51	Dom	Fig. S2d	Fig. S3f
		4	7.05 (G37)	3.70**	8.9	0.67–10.0	Heterozygous	OD		Fig. S3g
Branching (763 days)	143	1	25.0	3.68**	11.2	0.0–25.09	CV	Dom	Fig. S2e	Fig. S3h
Total seeds, year 3	140	10	29.0	3.81**	11.7	0.0–32.2	CV	Dom	Fig. S2f	Fig. S3i
Oil content, year 2	142	4	32.0	4.73***	13.3	2.0–34.3	G51	Dom	Fig. S2g	Fig. S3j
		10	31.0 (JCT27)	4.31***	12.1	4.0–32.2	G51	Dom		Fig. S3k
Oil content, year 3a	132	4	45.5	3.27**	10.8	0.0–57.1	G51	Dom	Fig. S2h	Fig. S3l
Oil content, year 3b	112	10	32.0	3.05*	11.8	1.0–32.2	G51	Dom	Fig. S2i	Fig. S3m
100-seed weight, year 2	142	4	7.05 (G37)	7.90***	22.6	1.3–15.0	G51	Dom	Fig. S2j	Fig. S3n
100-seed weight, year 3a	132	4	1.34 (1407326|12327601)	5.04***	16.1	0.0–19.0	G51	Dom	Fig. S2k	Fig. S3o
100-seed weight, year 3b	112	4	4.0	3.44**	13.2	0.0–52	G51	Dom	Fig. S2l	Fig. S3p

^a^The LOD significance thresholds are *** *p* = 0.01, ** *p* = 0.05 or * *p* = 0.10

^b^Effects are overdominant (OD), additive (Add) or dominant (Dom)

### Identification of QTL for seed number per plant, seed weight and oil content in mapping population G51 × CV

For the second harvest year after transplantation, although we observed a large variation in the number of seeds produced per plant (Additional file 4: Figure S1i), we did not observe any QTL associated with this trait. For the third harvest year, a single QTL was observed on linkage group 10, which accounted for an estimated 11.7 % of the phenotypic variance (Table 3; Fig. 4). This QTL was dominant, with the CV allele being beneficial compared to the G51 allele. Interestingly, an oil content QTL was also observed at a similar position on linkage group 10 for the second harvest year and the second harvest of year 3, accounting for between 11.8 and 12.1 % PVE. This QTL was dominant, with the beneficial allele being from the G51 parent (Additional file 6: Figures S3j, m). Although this may suggest that there is a potential reduction in oil content in response to a higher level of seed production, it should be noted that no correlation was observed for seed number and oil content in the second harvest year, and the correlation was weak but positive in the third harvest year (Table 2). A further QTL for oil content was observed in the second harvest year on linkage group 4. This locus was dominant and accounted for 13.3 % PVE. The beneficial allele was from the G51 parent. A QTL at a similar position was also identified for the first (but not second) harvest of year 3 (PVE = 10.8 %).

### QTL contributing to fatty acids composition of mapping population G51 × CV

In *J. curcas*, the two main fatty acids present in the storage oil are oleate and linoleate. For biodiesel production, monounsaturated fatty acids such as oleate are regarded as being desirable, as they have greater oxidative stability than polyunsaturated fatty acids and do not have poor cold-flow and cloud-point characteristics associated with saturated fatty acids [1, 25, 26]. It has been shown previously that plant growth temperature is likely to play a significant role in the proportion of these two fatty acids [1]. Within this mapping population we also found a strong negative correlation in the percentage of oleate (42.6–50.5 %) and linoleate (26.6–35.3 %) content within the seeds, suggesting that variation in these two fatty acids is both genetically and environmentally determined (Table 4 and Additional file 6: Figure S1). A number of QTL were observed for these two fatty acids (Table 5). On linkage group 6, a QTL was observed at 2 cM (10.8 % PVE) and 3 cM (11.9 % PVE), respectively, for oleate and linoleate content. Given the strong negative correlation between these two fatty acids, it is probable that the same underlying gene is responsible. Two additional QTL for linoleate content were observed on linkage groups 4 (at 4 cM) and 8 (at 11.5 cM), with PVE of 11.1 and 9.9 %, respectively.

**Table 4:**
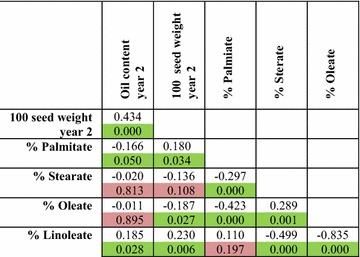
Pearson correlation coefficients for oil content, 100-seed weight and fatty acid composition in the mapping population G51 × CV

The upper uncoloured cells contain the *R* values. The lower coloured cells contain the *p* values. Cells shaded in green represent correlations with a *p* value <0.05 and cells shaded in red represent a *p* value >0.05. Details of data collection and calculation for each trait are provided in “Methods”

**Table 5:** Summary of QTL observed for fatty acid composition mapping population G51 × CV

Trait	Observations (n)	Method	Linkage group	Position (cM)	LOD^a^	PVE	Bayes 95 % CI (cM)	“High” genotype	Effect^b^	QTL plot Additional file 5:	Effect plot Additional file 6:
% Palmitate	140	HK	5	28.0	5.48***	13.2	19.2–41.6	CV	Add	Fig. S2m	Fig. S3q
		HK	7	58.0	3.36**	7.8	45.0–73.5	CV	Rec		Fig. S3r
		HK	10	32.0	3.12*	7.3	0.0–32.2	Heterozygous	OD		Fig. S3s
% Stearate	140	HK	7	25.0	8.34***	16.1	13.0–31.0	G51	Add	Fig. S2n	Fig. S3t
		HK	4	27.0	6.01***	12.3	23.0–39.0	CV	Add		Fig. S3u
		HK	8	11.0	5.34***	10.9	2.0–21.0	G51	Dom		Fig. S3v
		HK	1	9.9 (1398420|12336456)	3.57**	6.4	2.0–25.1	G51	Dom		Fig. S3w
% Oleate	140	HK	6	2.0	3.47**	10.8	2.0–11.0	CV > G51 > Het	−ve, OD	Fig. S2o	Fig. S3x
% Linoleate	140	HK	6	3.0	5.05	11.9	0.0–7.0	G51	Dom	Fig. S2p	Fig. S3y
		HK	4	4.0	4.75	11.1	0.0–36.0	G51	Dom		Fig. S3z
		HK	8	11.5 (JCT23)	4.26	9.9	2.0–27.0	CV	Dom		Fig. S3aa

^a^The LOD significance thresholds are *** *p* = 0.01, ** *p* = 0.05 or * *p* = 0.10

^b^Effects are overdominant (OD), additive (Add) or dominant (Dom)

The two other main fatty acids present in the seeds of *J. curcas* are palmitate (10.7 %–13.9 %) and stearate (6.1–9.2 %). Although the variations in stearate content were minor, four QTL were detected for stearate (Table 5), accounting in total for 45.7 % PVE. One of these mapped to a similar position as the linoleate QTL on linkage group 8. Three QTL were observed for palmitate content, accounting for 28.3 % PVE in total (Table 5).

### Identification of QTL for seed number per plant, seed weight and oil content in mapping population G33 × G43

Mapping population G33 × G43 was originally developed for the purpose of identifying a locus responsible for the biosynthesis of phorbol esters [14], the principal toxin in *J. curcas* seeds. However, we were also able to identify a number of QTL for seed traits using this population (Table 6; Additional file 7: Figure S4, Additional File 8: Figure S5 and Additional file 9: Figure S6). Pearson correlation analysis of the trait data (Table 7) revealed that for all 3 years, the calculated oil yields were mainly dependent on the number of seeds produced per plant (*R* ≥ 0.98 for all 3 years). Weak, but significant correlations were observed for oil content and oil yields in years 1 and 3 (*R* = 0.333 and 0.123, respectively), but not in year 2. Interestingly, weak but significant correlations between 100-seed weight and oil yield were observed for all three years, but these were positive in year 1 (*R* = 0.203) and year 2 (*R* = 0.316) but negative in year 3 (*R* = −0.142). Similarly, a negative correlation was observed between the 100-seed weight and number of seeds produced per plant during year 3 (*R* = −0.273). This may indicate that in the third year for this mapping population, source strength rather than sink capacity is important (i.e. as the plants produce more seeds, they are able to allocate fewer resources per seed), or that there is greater competition between individual plants of the mapping population for light or nutrients as the size of the plants increase.

**Table 6:** Summary of QTL observed for mapping population G33 × G43

Trait	Observations (n)	Linkage group	Position (cM)	LOD^a^	PVE	Bayes 95 % CI (cM)	Beneficial allele	Effect^b^	QTL plot Additional file 8:	Effect plot Additional file 9:
Total seeds Year 2 (NP^c^)	254	5	22.9 (G104)	3.05*	4.6	0.0–37.0	G33	Dom	Fig. S5a	Fig. S6a
Total seeds Year 3	251	7	30.3 (1398222|12344025)	3.52**	5.9	12.0–38.0	G33	Dom	Fig. S5b	Fig. S6b
		4	8.0	3.19*	5.4	0.0–34.5	G33	Dom		Fig. S6c
100-seed weight Year 1	243	4	40.0	6.12***	9.5	28.0–55.0	G33	Rec	Fig. S5c	Fig. S6d
		2	28.0	5.66***	8.7	24.3–35.0	G33	Dom		Fig. S6e
		11	20.5 (G28)	4.16***	6.3	4.0–37.0	G33	Add		Fig. S6f
100-seed weight Year 2	250	4	35.9 (AG806)	15.09***	18.5	30.0–42.0	G33	Add	Fig. S5d	Fig. S6g
		11	26.0	11.39***	13.5	21.2–27.0	G33	Add		Fig. S6h
		2	23.0	5.35***	6.0	18.0–26.0	G33	Dom		Fig. S6i
		10	3.0	4.39***	4.9	0.0–11.0	G43	Dom		Fig. S6j
100-seed weight Year 3	253	4	41.0	6.82***	8.9	35.9–58.9	G33	Add	Fig. S5e	Fig. S6k
		9	50.8 (1408478|12317739)	4.41***	5.6	42.0–51.5	G33	Dom		Fig. S6l
		11	1.0	3.53***	4.5	0.0–10.0	G33	Rec		Fig. S6m
		2	37.0	3.15**	4.0	15.0–40.0	G33	Dom/OD		Fig. S6n
		11	26.8 (1398998|12324489)	2.96**	3.7	7.0–32.0	G33	Add		Fig. S6o
		10	4.0	2.64**	3.2	0.0–15.0	G43	Dom		Fig. S6p
Oil content Year 2	249	6	24.0	5.62***	7.6	1.0–34.0	G43	Rec	Fig. S5f	Fig. S6q
		10	0.0 (Jcuint152)	4.89***	6.5	0.0–5.0	G43	Add		Fig. S6r
		4	2.5 (1416680|12276533)	4.51***	6.0	0.0–10.0	G33	Dom		Fig. S6s
		5	43.8 (AG517)	4.13***	5.5	27.7–49.0	G33	Dom		Fig. S6t
Oil content Year 3	253	5	48.0 (1407850|12321642)	7.07***	11.5	40.0–51.0	G33	Dom	Fig. S5g	Fig. S6u
		6	45.5 (1406827|12348279)	3.13***	4.9	1.0–51.0	G43	Rec		Fig. S6v

^a^The LOD significance thresholds are *** *p* = 0.01, ** *p* = 0.05 or * *p* = 0.10

^b^Effects are overdominant (OD), additive (Add) or dominant (Dom)

^c^QTL for total seed yield in year 2 were obtained by performing non-parametric analysis

**Table 7:**
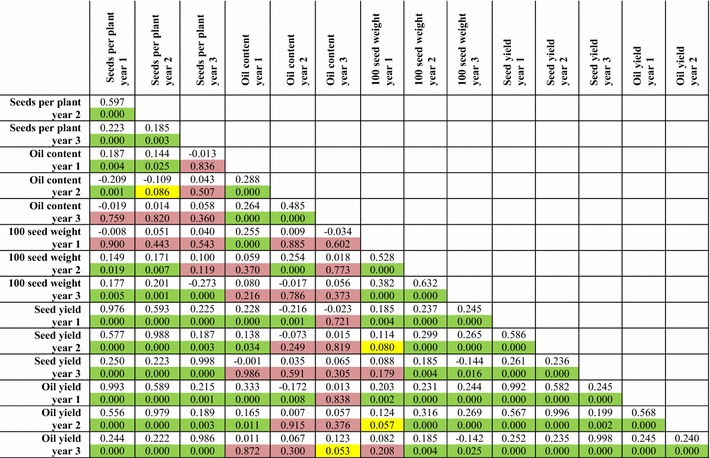
Pearson correlation coefficients for seed traits in mapping population G33 × G43

The upper uncoloured cells contain the *R* values. The lower coloured cells contain the *p* values. Cells shaded in green represent correlations with a *p* value <0.05, cells shaded in yellow represent a *p* value of between 0.05 and 0.10, whereas cells shaded in red represent a *p* value >0.10 (non-significant). Details of data collection and calculation for each trait are provided in “Methods”

For the first year we did not detect any QTL relating to the number of seeds per plant. For the number of seeds produced per plant during the second year, a weak QTL was observed (*p* < 0.10) when non-parametric analysis was performed. It should be noted, however, that the average number of seeds harvested per plant declined between years 1 and 2, due to adverse weather conditions at the field site of the G33 × G43 mapping population (see “Methods” and Additional file 7: Figures S4a, f). In the year 3, we observed that two QTL were found on linkage groups 4 and 7, accounting for 11.3 % PVE. The largest QTL detected for this population were for the 100-seed weights. In the first harvest year, three QTL were detected on linkage groups 2, 4 and 11, which accounted from 24.5 % PVE. In the second harvest year, three QTL at similar positions were also identified, alongside an additional QTL on linkage group 10. In total, these accounted for 42.9 % PVE. In the third year, six QTL for 100-seed weight were observed, although the total PVE declined to 29.9 %. The two additional QTL were on linkage group 9 and the upper arm of linkage group 11. The QTL on linkage groups 4 and in the middle of linkage group 11 were additive, whereas those on linkage groups 2, 9 and 10 were dominant. The QTL on the upper arm of linkage group 11 (year 3 only) was recessive. With the exception of the QTL on linkage group 10, the allele from the G33 parent was beneficial in each case. Based on the confidence intervals, it does not appear that the QTL on linkage group 4 of this mapping population is co-located with the 100-seed weight QTL we observed in mapping population G51 × CV. For the second harvest year, four QTL accounting for a total of 25.6 % PVE were detected from seed oil content, on linkage groups 4, 5, 6 and 10. In the subsequent year, we only observed the QTL on linkage groups 5 and 6, which had a total PVE of 16.4 %. The beneficial allele for the QTL on linkage groups 4 and 5 was from patent G33, whereas the beneficial allele for the other two QTL (linkage groups 6 and 10) were from parent G43. Two of these QTL, on linkage groups 4 and 10, may be related to the oil QTL observed in mapping population G51 × CV, though due to the relatively large QTL intervals compared to those observed in the G33 × G43 population, this would require further experimental confirmation. Interestingly, the oil content QTL on linkage group 10 also maps to a similar position as the seed weight QTL on this linkage group and in both instances, the G43 parent contributed the beneficial allele.

### Comparison of QTL positions with mapped candidate genes for lipid biosynthesis

Where the position of candidate genes are known, it is possible to compare QTL positions to determine whether they may potentially underlie a specific QTL. This approach is most effective when the confidence intervals for the QTL are low. Based on our successful mapping of the majority of the candidate genes we identified involved in lipid biosynthesis (Fig. 3 and Additional file 3: Table S14), we compared the positions of these genes and QTL. In mapping population G51 × CV the majority of the QTL had very large 95 % confidence intervals, but the main QTL for oleate and linoleate appeared to be located between 2.0 and 7.0 of linkage group 6 (Table 5).

A likely candidate gene for this QTL would be oleate desaturase (FAD2), an enzyme which converts an oleate group at the sn2-position of phospholipids to linoleate (Fig. 3, step 19). In *J. curcas* there are two FAD2 genes, both of which are expressed within developing seeds [27]. We mapped these to linkage groups 1 and 6 (Additional file 3: Table S3). The Bayes 95 % confidence intervals for the QTL would indicate that it is unlikely that the FAD2 on linkage group 6 could be the locus underlying the main QTL for oleate. However, the 95 % confidence intervals indicated that this QTL mapped between two markers (SNP12983 and 1406628|12346310) which both resided on a single 3.37 Mbp scaffold (KK915213.1) of the *J. curcas* genome sequence released by the Chinese Academy of Sciences (Additional file 2: Table S8). This scaffold contains 560 predicted gene sequences, of which 134 are located within the 726 kb of sequence between these two markers. Further analysis of polymorphisms in this region should provide more insight into discovering the underlying genetic basis of the observed variation between oleate and linoleate content. The strongest QTL for stearate content on linkage group 7 mapped in close proximity to the genes for both acyl-ACP thioesterase (Step 12) and an acyl-CoA synthetase. The acyl-ACP thioesterase gene of linkage group 7 encodes the FatA type of enzyme (Additional file 2: Table S14), which typically displays a preference for oleoyl-ACP, whereas the FatB type typically show broader specificity including activity with saturated acyl-ACPs [28]. The long-chain acyl-CoA synthetases involved in activation of the export and activation of fatty acids from the plastids also show broad specificity [29]. Although the colocalization of these two genes with the stearate QTL is interesting from a biological perspective, given the relatively minor importance and the small amount of absolute variation in stearate content, we do not think this QTL warrants further investigation from a plant breeding perspective.

**Fig. 5:**
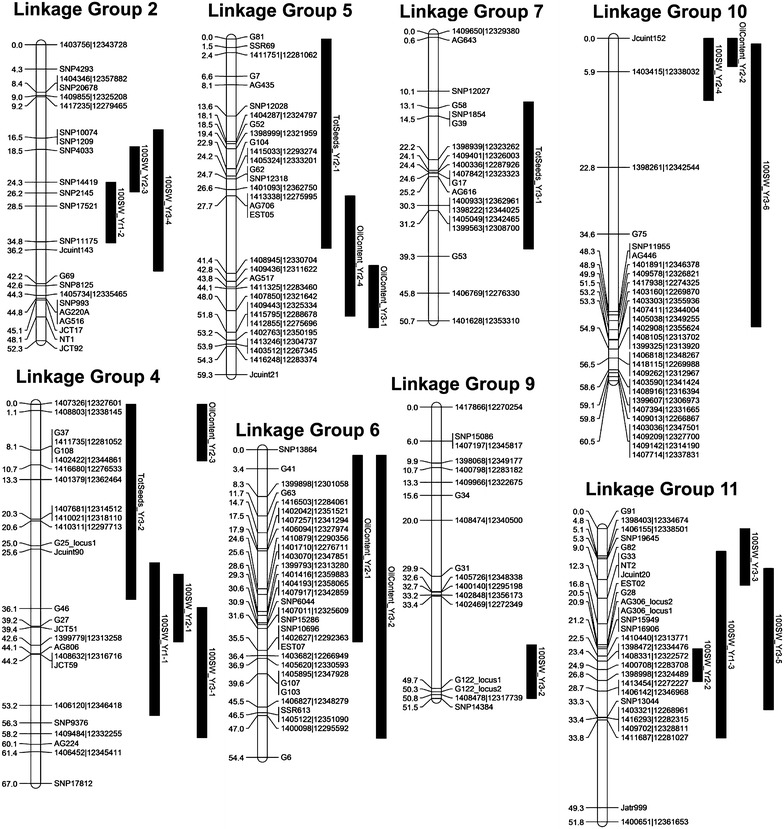
Map of QTL detected in mapping population G33 × G43. Only linkage groups found to contain QTL are shown

In the G33 × G43 mapping population, the QTL with the smallest interval was for oil content in the second harvest year. The Bayes 95 % confidence interval for this QTL indicated that it resided within a 5 cM interval on linkage group 10, between markers Jcuint152 and 1403415|12338032 (Additional file 2: Table S12). Both of these markers reside on a single 3.63 Mbp scaffold (KK914240.1) which contains 394 genes. It should be noted, however, that in comparison to the composite interval map (Fig. 2), 5 cM of the upper arm of the linkage group for mapping population G33 × G43 was not mapped and the QTL may have resided within this region. Interestingly, however, one of the candidate gene markers that mapped to scaffold KK914240.1 was for the *ABA Insensitive* (*ABI*) *4* gene. The ABI gene family includes abscisic acid (ABA)-responsive transcription factors which have roles in the regulation of a number of biochemical and developmental processes. In *Arabidopsis*, the ABI4 protein is known to be a regulator of *DGAT1* expression in seedlings [30]. The role of ABI4 in oil accumulation during seed development is less clear, and ABI3 seems to play a more dominant role [31]. The role of ABI genes in *Jatropha* has not been studied extensively, but ABI4 expression has been shown to correlate with the stages of seed development in which oil accumulation occurs [32]. The oil content QTL on linkage group 5, which appeared in both years 2 and 3, produced relatively short confidence interval of 11 cM (Table 6). Although this QTL interval could not be located to a single scaffold of the genome, analysis of the combined genetic/physical map (Additional file 2: Table S3) and the population-specific map for G33 × G43 (Fig. 5) revealed that 9 cM of this region corresponded to a single scaffold (GenBank KK914632.1, containing a predicted 133 genes). A pair of tandemly duplicated phosphatidate phosphatase (PAP) genes is located on this scaffold (Fig. 3, step 17 and Additional file 3: Table S14). The PAP enzyme is part of the ER pathway and converts phosphatidic acid into diacylglycerol. In *Arabidopsis*, a PAP gene was also shown to underlie a QTL for oil content in a mapping population segregating for this trait [33]. These two PAP genes in *J. curcas* therefore represent strong potential causal gene candidates responsible for the oil content QTL on linkage group 5. One further oil content QTL on linkage group 4 also had a relatively short confidence interval of 10 cM. Comparison of the marker positions (Fig. 5) with the mapped scaffolds indicated that this QTL is likely to reside on scaffold KK914227, which is 2.74 Mbp and contains 274 predicted genes (Additional file 2: Table S6). Included within these genes was one of the mapped lipid biosynthesis genes, malonyl-CoA:ACP malonyl transferase (Fig. 3 and Additional file 3: Table S6). Our future work will involve characterization of these genes in the different parental populations, including upstream regions and gene expression levels, to determine whether there is any variation between the two parental lines.

### Future approaches to QTL mapping in *J. curcas*

In addition to being able to identify a number of QTL, we were in some cases able to identify specific DNA scaffolds from the CAS *Jatropha* genome assemblies underlying these QTL and even identify candidate genes that may be responsible for these QTL. Nonetheless, in many instances, the QTL confidence intervals were too large to identify specific genome regions. The mapping resolution obtained by the family-based mapping approach is often limited as QTL intervals are usually dependent on population size, QTL effect and marker density [34]. Increasing the number of meioses within a mapping population by generating advanced-generation crosses can be used for finer mapping of QTL, but this approach is impractical with perennial plants because of the length of time required to produce and collect phenotypic data from each generation. An alternative approach that improves the ability to identify loci-controlling traits is a genome-wide association study (GWAS). This approach permits a higher resolution than family-based mapping by exploiting historical recombination events and does not therefore rely on the creation of experimental populations. The use of germplasm collections rather than biparental crosses also permits the identification of beneficial alleles from a wider genetic background. We believe that the advances that have been obtained by combined genetic and physical mapping that have been reported in the current study and elsewhere [18], together with the improvements in our knowledge of the availability of genetically diverse germplasm for this species within Mesoamerica [10, 12], make GWAS a feasible next step. In addition, it should also be possible to further improve and integrate the genetic and physical maps of *J. curcas* by developing molecular markers for unmapped scaffolds using an approach similar to the one we used previously to fine-map the phorbol ester biosynthesis locus in *J. curcas* [14]. These approaches should lead to the identification and characterization of a greater number of QTL from a wider genetic pool.

## Conclusions

The identification of QTL for traits associated with oil yield in two mapping populations of *J. curcas* is a significant step forward in the development of improved commercial varieties of *J. curcas*. By stacking a number of these QTL, together with the locus we previously identified controlling phorbol ester biosynthesis [14], it should be possible to create higher-yielding non-toxic varieties suitable for the production of both vegetable oil and seed meal that can readily be converted into animal feed. The use of marker-assisted breeding is particularly beneficial for a large perennial plant such as *J. curcas*, as it allows selection of individuals containing multiple beneficial alleles prior to transplantation from nursery to the field. For QTL which are additive or dominant, the implementation of a breeding strategy would involve creating genetically stable (near homozygous) plants. Ordinarily, in plant breeding, the aim is to introgress one or more QTL into an “elite” cultivar and then remove non-target regions through successive backcrossing. Due to the present lack of such elite cultivars in *J. curcas*, it is instead likely that the approach adopted would require a combination of phenotypic and genotypic selection to ensure that new lines are both genetically stable and display superior performance compared to existing varieties, i.e. in the absence of any other supporting information, non-QTL regions could contain homozygous background from either parental plant.

One of the most interesting QTL to be identified from this study was a pleiotropic QTL on linkage group 4 which contributed to both plant height and stem diameter, both of which were shown to correlate positively with oil yield (*R* = 0.306–0.396, Additional file 2: Table S2). The fact that these QTL were overdominant indicates that heterosis (i.e. use of F_1_ hybrids) may be an effective strategy in the development of new varieties of *J. curcas*. As discussed previously, implementation of this approach would require a method of producing F_1_ plants on a large scale. Nonetheless, a further investigation into the potential of heterosis in *J. curcas* could be evaluated by first identifying or creating near-isogenic parental lines from the diverse germplasm that is found in Mesoamerica.

In summary, the QTL identified in this study provide a valuable starting point for the development of new cultivars of *J. curcas*. In conjunction with phenotypic selection, these markers can be used to create genetically stable cultivars containing multiple QTL that are likely to improve the overall yield of this important emerging oil crop.

## Methods

### Mapping populations

The two F_2_ mapping populations used for QTL analysis have been described previously [14]. Mapping population G51 × CV was grown at (13°57′33.17″N and 90°23′21.89″W) and transferred from the nursery to the field on 25 May 2010. Mapping population G33 × G43 was grown at (13°57′41.18″N and 90°23′29.77″W) and transferred from the nursery to the field on 23 July 2011. Both mapping populations were grown at a density of 4 m by 2 m (equivalent to 1250 plants per hectare). The transplantation of both populations was done during the rainy season in Guatemala (May–October). During the dry season (November–April), the plants were watered with a drip irrigation system. Fertilization was done through the irrigation system according to the nutritional requirements of the plant and soil analyses.

### Genotyping and linkage map construction

The development of molecular markers and construction of genetic linkage maps for the populations used in this study have been described previously [14, 35]. Additional SSR markers were added to the map, either to fill in gaps or locate the position of specific candidate genes. The sequences of these SSR markers are provided in Additional file 1: Table S1. A list of markers linked to candidate genes involved in oil biosynthesis [27, 36] is provided in Additional file 3: Table S14.

### Collection of phenotypic data

Plant heights, stem diameters, canopy diameters and the number of branches per plant were recorded at specific dates after transplantation as detailed in Table 1. For canopy areas, two measurements were taken: the first measurement was taken along the axis of the row (2 m plant spacing), whereas the second measurements were taken on the axis between rows (4 m plant spacing). These values were then used to calculate the canopy areas using the formula CA = *π* × *r*1× *r*2. The total number of seeds collected per harvest year was calculated from 1 February to 31 January. Oil content and seed weights were determined using an Oxford Instruments MQC Benchtop NMR analyser (Abingdon, Oxfordshire) [37]. The machine was calibrated for oil content using pre-weighed samples of pure *Jatropha* oil in glass vials. For calibration of water content, samples of seeds which had been stored at ambient temperature and different relative humidities were used. For each plant, typically 48 seeds, but minimally 20 seeds, were used to determine the oil and moisture content. Oil contents and 100-seed weights were then calculated by adjusting the values for all samples to 7 % water. Seed yields were calculated by multiplying the total number of seeds per plant by the 100-seed weight/100. This oil yield was calculated by multiplying seed yield by the percentage oil content/100. To analyse fatty acid compositions, 24 seeds were ground to a fine powder using a domestic coffee grinder. A small aliquot (ca. 10 mg) of the ground seed was then converted to fatty acid methyl esters and analysed on a gas chromatograph equipped with a flame-ionization detector as described previously [38].

### QTL analyses

After construction of the genetic maps, non-segregating markers were binned to form a single marker. Where possible, gaps in the map were filled using information from flanking markers. Finally, a number of markers which were only partially informative were removed. The resulting datasets are provided as Additional files 10 and 11. QTL analysis was performed using R/qtl [39]. An initial scan was performed using Haley–Knott regression [40]. LOD thresholds were determined using 10,000 permutations, and significance thresholds were set at *p* = 0.10, *p* = 0.05 and *p* = 0.01. After the identification of the initial QTL, Haley–Knott regression analysis was performed using the *makeqtl* and *addqtl* functions. This process was repeated until no further QTL with LOD scores corresponding to *p* = 0.1 were observed. Two-dimensional, two-QTL scans were also performed using the *scantwo* function, using significance thresholds determined from 1000 permutations, but these did not reveal any additional QTL. The QTL positions were then refined using the *fitqtl* command, which also provided estimates of the percentage of phenotypic variation explained by each QTL. Interval estimates (95 % confidence) of QTL locations were obtained using the Bayes credible interval function (*bayesint*). For datasets displaying non-normal distributions, non-parametric tests were also performed. However, only one additional QTL was detected using this method (total seeds in year 2 for mapping population G33 × G43, Table 6). Finally, composite interval mapping was also performed using a window size of 10 cM, using three markers as co-variables. The outputs from these analyses are included within the plots for the QTL analyses shown in Additional file 5: Figure S2 and Additional file 8: Figure S5. The QTL effects (additive, dominant or overdominant) and source of the parental source of the beneficial alleles were determined by ANOVA analysis of the genotype versus phenotype at the QTL position, in conjunction with post hoc analysis using Tukey’s test (Additional file 6: Figure S3 and Additional file 9: Figure S6).

## Additional files

**Additional file 1: Table S1.** Sequences for new SSR markers added to the linkage map
**Additional file 2: Table S2.** Summary of genome sequence physically mapped onto the *Jatropha curcas* genetic map shown in Figure 1. Tables S3–S13. Summary of GenBank contigs mapped onto Groups 1 to 11 of the *Jatropha curcas* linkage map
**Additional file 3: Table S14.** Mapping of genes involved in seed oil biosynthesis onto the *Jatropha curcas* linkage map
**Additional file 4: Figure S1.** Distribution of trait data recorded for mapping population G51 × CV
**Additional file 5: Figure S2.** Initial QTL scans produced for traits recorded for mapping population G51 × CV
**Additional file 6: Figure S3.** Boxplots of genotype versus phenotype for QTLs in mapping population G51 × CV
**Additional file 7: Figure S4.** Distribution of trait data recorded for mapping population G33 × G43
**Additional file 8: Figure S5.** Initial QTL scans produced for traits recorded for mapping population G33 ×  G43
**Additional file 9: Figure S6.** Boxplots of genotype versus phenotype for QTLs in mapping population G33 x G43
**Additional file 10:** Phenotype and genotype date from mapping population G51 × CV
**Additional file 11:** Phenotype and genotype date from mapping population G33 × G43

## Abbreviations

ABA: abscisic acid
ABI: ABA insensitive
ACP: acyl carrier protein
AFLP: amplified fragment length polymorphism
ANOVA: analysis of variance
CAS: Chinese Academy of Sciences
cM: centimorgan
CoA: coenzyme A
DGAT: diacylglycerol acyltransferase
ER: endoplasmic reticulum
FAD: fatty acyl desaturase
GWAS: genome-wide associated study
LOD: logarithm of odds
Mbp: megabase pairs
NMR: nuclear magnetic resonance
PAP: phosphatidate phosphatase
PVE: phenotypic variance explained
QTL: quantitative trait locus/loci
SNP: single nucleotide polymorphism
SSR: simple sequence repeat
