# Supplementary material for: Identification of QTL markers contributing to plant growth, oil yield and fatty acid composition in the oilseed crop Jatropha curcas L
Source: Biotechnol Biofuels. 2015 Sep 25;8:160. doi: 10.1186/s13068-015-0326-8 (PMC4583170; doi:10.1186/s13068-015-0326-8)
Supplement: Supplementary file 1 — Additional file 1: Table S1. Sequences for new SSR markers added to the linkage map [file 13068_2015_326_MOESM1_ESM.docx]

**Addition File 1: Table S1 – Sequences for new SSR markers added to the linkage map**

| **Marker** | **M13-tagged primer** | **Non-tagged primer** | **SSR** |
| --- | --- | --- | --- |
| ***Linkage group 1*** | | | |
| G427 | 5’-[M13]-GAAGCCTGTCCTGCCGCGAT-3’ | 5’-AGGCTTGAGAAGTTGAGTGCCCA-3’ | (AT)_34_ |
| ***Linkage group 2*** | | | |
| G351 | 5’-[M13]-CCCCAATCTGGCCATCCGCC-3’ | 5’-GGGTGGAAGCAATCATAGGAAAAGGA-3’ | (GA)_16_ |
| G416 | 5’-[M13]-gttcggtcctccgtcatgcga-3’ | 5’-tcggagccgccattctcacac-3’ | (TC)_18_ |
| G410 | 5’-[M13]-CCAGGCTGAGTACCGGCTGGA-3’ | 5’-TGGCTGACTCTGATGATGTCAGTT-3’ | (TC)_13_ |
| G382 | 5’-[M13]-ACCCATCCCCTCACACGGCA- 3’ | 5’-TCTGTCCTTGTCCTTGTCCTGCT-3’ | (AT)_13_ |
| G437 | 5’-[M13]-AGGACCACCATGCACTGCCA-3’ | 5’-AGAAACCCCATTTCTAAGCAATTGAA-3’ | (AT)_19_ |
| ***Linkage group 3*** | | | |
| G125 | 5’-[M13]-ACGGCGTAGACTAGAAAGAAAGCACA-3’ | 5’- ACCAAAGCTGAACAAATTTTATGTTGGT-3’ | Imperfect |
| G439 | 5’-[M13]-TGTGTCCTTAGCTTGATGGACTTGG-3’ | 5’-CGAGCTAAAAAGGAACTGCCTTGGG-3’ | (ATT)_13_ |
| G126 | 5’-[M13]-TCCCAAAAAGCCTACCCCCAGC-3’ | 5’-TGGTGGTAGTGGAGTCGTGTTTGA-3’ | (GTG)_5_….(GGT)_5_ |
| G55B | 5’-[M13]-ATGCACAGCCTTGGCAACAC-3’ | 5’-TCGTACCCATCTGTCAGGTCAGTTT-3’ | (TC)_25_ |
| G434 | 5’-[M13]-TCCTCCCAAACCAACGTGGA-3’ | 5’-CCCTTCAATTTGGTTAAAAATGTGTGT-3’ | (AT)_16_ |
| G423 | 5’-[M13]-GCTTTGCCCTTTTTGGTATTTTCTCCC-3’ | 5’-CGGTCCCCTAAATTCTCATGTGCTGC-3’ | (ATA)_11_…(AAAT)_6_ |
| G386 | 5’-[M13]-AGGCATGTGGTGAGAAGTGGA-3’ | 5’-TCAGGATAAGCATACCACTCAGTCA-3’ | (AT)_23_ |
| G436 | 5’-[M13]-ACCTGCTGCAGCCTGAAAACT-3’ | 5’-TGGCCGACATCTTGGCACAGG-3’ | (AT)_19_ |
| ***Linkage group 4*** | | | |
| G407 | 5’-[M13]-AGTATTGTTTTTAACCTATCCGACACT-3’ | 5’-TGCGGTGAAATTATTGGTTAGGCCTTT-3’ | (ATA)_10_ |
| G357 | 5’-[M13]-ACCAAAAGGCAAACGAAGCACCC-3’ | 5’-TTGGTCAATTAAGAGGAGGGCTACA-3’ | (AT)_18_ |
| G352 | 5’-[M13]-TTTTTCCCCCAACACACCAC-3’ | 5’-GCATGGAGCGGTTTGGTTATGGT-3’ | (TA)_12_ |
| G376B | 5’-[M13]-AGCGACCATCAAACCATGCCA-3’ | 5’-GGATGATGATGAGTACGTAGGAAACC-3’ | (AT)_12_ |
| ***Linkage group 5*** | | | |
| G255 | 5’-[M13]-TGAAGTGTTGGCCCCACGTA-3’ | 5’-AGCCTAAGTCTGGTGCTCTATTTACT-3’ | (TC)_5_(TA)_11_ |
| G415 | 5’-[M13]-TGTCCCCTTCCCTAGGCCAT-3’ | 5’-TGCCCAAAAATGTAACTCCACTCAA-3’ | (TA)_24_ |
| ***Linkage group 6*** | | | |
| G411 | 5’-[M13]-TGACTCGGAGCTACAAGCCTACA-3’ | 5’-GCAGAGGCAAGAGCTAGGCTGC-3’ | (TA)_12_(TTTA)_4_ |
| G394 | 5’-[M13]-TCTGCAGTGCAAATATAAACCTCGC-3’ | 5’-AGCTCTGATCGGCAATAAAACACA-3’ | (TG)_6_(TA)_22_ |
| G390 | 5’-[M13]-TGCATGGCTAGCGGTGATGGA-3’ | 5’-ACCATGTCACCCCCTCCTTCCT-3’ | (GA)_15_ |

**Addition File 1: Table S1 continued – Sequences for new SSR markers added to the linkage map**

| **Marker** | **M13-tagged primer** | **Non-tagged primer** | **SSR** |
| --- | --- | --- | --- |
| ***Linkage group 8*** | | | |
| G139D | 5’-[M13]-CGGACAGGAACCCACCGACC-3’ | 5’-TGCCTCTCAAGGATGTGGAGCA-3’ | (AG)_12_ |
| G139E | 5’-[M13]-AGCTGGTCGGTTGGTCCCCT-3’ | 5’-AGGTCATCGACGGGTGGTCGT-3’ | (GCTGTG)_5_ |
| G440 | 5’-[M13]-TGCGCAATCAGCTACACCAAGC-3’ | 5’-GGTCGCAGTGGTACACCGTGG-3’ | (CT)_9_CC(CT)_15_ |
| G276B | 5’-[M13]TCCGCTATGCCACGATCCAA-3’ | 5’-AGCCTCTGGGTCCAACAGTGA-3’ | (AC)_12_ |
| G368 | 5’-[M13]-ACGCTGGCTCCAATTTTCCCA-3’ | 5’-TGCACCTTAACGACAGAAACGCA-3’ | (TA)_15_ |
| G377 | 5’-[M13]-TGGCTTTGCACTAACACAGGGC-3’ | 5’-AGTCATGATCACTTTCCACCATTAAA-3’ | (CA)_24_(TA)_6_(GT)_6_ |
| G268 | 5’-[M13]-CGGCGATCCAGATGCTGCTGA-3’ | 5’-TCTGACAGGTCACCAGAAAGGCT-3’ | (AT)_19_ |
| G269 | 5’-[M13]-GCCAGGCGAGAGGTTGGAGG-3’ | 5’-ACCAGATTTTTCCCACCATGCATACCA-3’ | (AAT)_8_(TAT)_6_(ATT)_8_ |
| G261 | 5’-[M13]-AGCAACTAAAATCCTCGGGGAGA-3’ | 5’-TGCGCCTAATGAATTGATAACTGTCT-3’ | (AT)_21_ |
| ***Linkage group 9*** | | | |
| G3 | 5’-[M13]-TCGGTTCTTGATGAGCTATTGCAGAC-3’ | 5’-CACTTCAATTGCCCGAAACAAAATTGC-3’ | (TTC)_11_ |
| G413 | 5’-[M13]-CCTGGCTACCTACACCAGAACACG-3’ | 5’-ACAAGGTTGCAGGTCAGAAAAGATTGT-3’ | (TA)_20_ |
| G354 | 5’-[M13]-GGCACGAGATGAGTTTGCTTCTGC-3’ | 5’-TCGATGGTGACTGAATGAACGAAGT-3’ | (AT)_12_ |
| G378 | 5’-[M13]-TCTCATTTTTCTCCCTTCTTTCCCAC-3’ | 5’-GAAAATTGACGCAAAAGATTGAGAACG-3’ | (TAT)_21_…(TAT)_11_ |
| ***Linkage group 10*** | | | |
| G373B | 5’-[M13]-ACATTTAGGTTACGTAGACGCCAT-3’ | 5’-TGGATTCCACCAACTGAGTTATTTGT-3’ | (TA)_12_ |
| G236 | 5’-[M13]-TGCTTTGCTTGTCACCACAT-3’ | 5’-TCATTATTCTCAATCCATACTCTTGA-3’ | (TAA)_9_ |
| G235 | 5’-[M13]-AGAGCGTCGGGTCAACATCC-3’ | 5’-AGCATTGGAAAGGGAAAGAAAAGGA-3’ | (TA)_16_ |
| G238 | 5’-[M13]-AGCAAGAGTCCCCCAACAGT-3’ | 5’-ACCGACCAACCGAAAAGTCGCC-3’ | (AT)_20_ |
| G240 | 5’-[M13]-GCCGCCTCCTTGCTCTTCCC-3’ | 5’-GGTGGTGTCGCGACGGAGAG-3’ | (GA)_17_ |
| G247 | 5’-[M13]-ACATGGAGCCTCCGACAACA-3’ | 5’-TGGAAACACATACTCGTGGAGCA-3’ | (TTA)_9_ |
| ***Linkage group 11*** | | | |
| G163 | 5’-[M13]-CTTCTGGTGTCACGATGGTC-3’ | 5’-TGTTTTCAAGTCATTTTCACGTTT-3’ | (AT)_15_ |
| G226 | 5’-[M13]-CCGCGTCTCCAGGCATGGTT-3’ | 5’-TGTGGCAAAAGCAAGGAAGAACA-3’ | (AT)_20_ |
| G388B | 5’-[M13]-TGTCCAGAAGCATCTACACCCTCG-3’ | 5’-AGGTTATGAGACCAGCCATTGAAAGT-3’ | (AT)_5_A(AT)_7_ |
| G401 | 5’-[M13]-TCACGGTTTCCTGTTTCCTCCTCA-3’ | 5’-GATTGGATTCAAATTCTCTTCAAGCGG-3’ | (TA)_20_ |
| G414B | 5’-[M13]-ACCACTCTACCACCACCACCA-3’ | 5’-TGGCCAAACCGAATGCTCACCC-3’ | Imperfect |
