# Supplementary material for: Identification of QTL markers contributing to plant growth, oil yield and fatty acid composition in the oilseed crop Jatropha curcas L
Source: Biotechnol Biofuels. 2015 Sep 25;8:160. doi: 10.1186/s13068-015-0326-8 (PMC4583170; doi:10.1186/s13068-015-0326-8)
Supplement: Supplementary file 4 — Additional file 4: Figure S1. Distribution of trait data recorded for mapping population G51 × CV [file 13068_2015_326_MOESM4_ESM.docx]

**Additional File 4: Figure S1 – Distribution of trait data in mapping population G51 x CV**

**Figure S1:** Distribution of phenotypic traits in mapping population G51 x CV including **(a)** plant height at 567 days after transplantation, **(b)** plant height at 763 days, **(c)** stem diameter (at base of plant) at 567 days, **(d)** stem diameter (at base of plant) at 763 days, **(e)** canopy area at 567 days and **(f)** canopy area at 763 days.

**Additional File 4: Figure S1 continued – Distribution of trait data in mapping population G51 x CV**

**Figure S1 continued:** Distribution of phenotypic traits in mapping population G51 x CV including **(g)** number of branches at 567 days after transplantation, **(h)** number of branches at 763 days, **(i)** number of seeds produced per plant in the second year after transplantation, **(j)** number of seeds produced per plant in the third year after transplantation, **(k)** percentage oil content of seeds in second year after transplantation and **(l)** percentage oil content of seeds in third year (harvest 1).

**Additional File 4: Figure S1 – Distribution of trait data in mapping population G51 x CV**

**Figure S1 continued:** Distribution of phenotypic traits in mapping population G51 x CV including **(m)** percentage oil content of seeds in the third year after transplantation (harvest 2), **(n)** 100 seed weight in the second year, **(o)** 100 seed weight in the second year (harvest 1), **(p)** 100 seed weight in second year (harvest 2), **(q)** seed yield per plant in year 2 and **(r)** seed yield per plant year 3.

**Additional File 4: Figure S1 – Distribution of trait data in mapping population G51 x CV**

**Figure S1 continued:** Distribution of phenotypic traits in mapping population G51 x CV including **(s)** oil yield per plant in the year 2, **(t)** oil yield per plant in the year 3, **(u)** palmitate content as a percentage of total fatty acid content of seed oil from year 2, **(v)** stearate content as a percentage of total fatty acid content of seed oil from year 2, **(w)** oleate content as a percentage of total fatty acid content of seed oil from year 2 and **(x)** linoleate content as a percentage of total fatty acid content of seed oil from year 2.
