# Supplementary material for: Identification of QTL markers contributing to plant growth, oil yield and fatty acid composition in the oilseed crop Jatropha curcas L
Source: Biotechnol Biofuels. 2015 Sep 25;8:160. doi: 10.1186/s13068-015-0326-8 (PMC4583170; doi:10.1186/s13068-015-0326-8)
Supplement: Supplementary file 5 — Additional file 5: Figure S2. Initial QTL scans produced for traits recorded for mapping population G51 × CV [file 13068_2015_326_MOESM5_ESM.docx]

**Additional File 5 : Figure S2 - Initial QTL scans produced for traits recorded for mapping population G51 × CV**

**
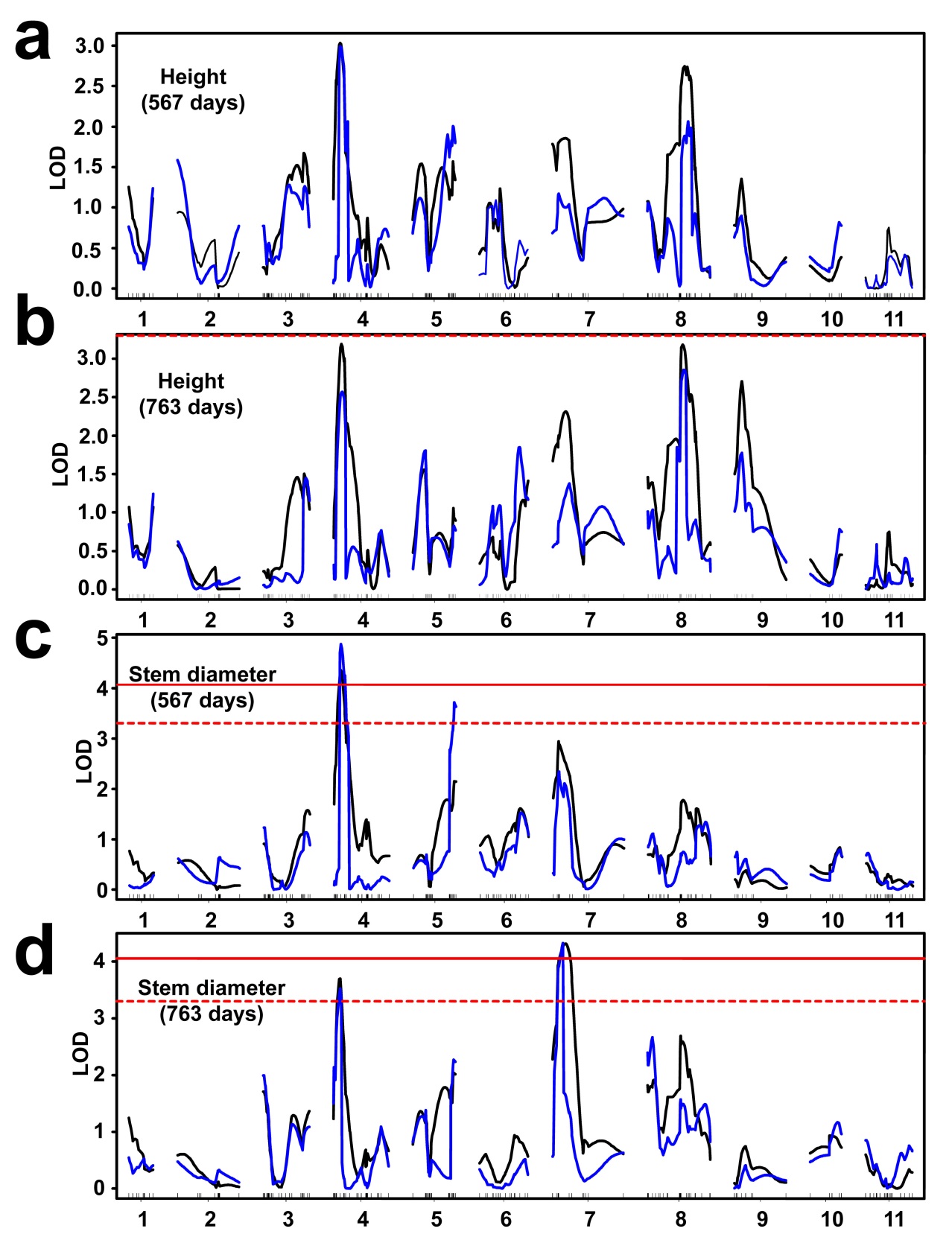
**

**Figure S2:** Outputs of initial QTL scans obtained using the Harley-Knott (black lines) and composite interval mapping (blue lines) in R/qtl. The lower dashed red line indicates the significance threshold at *p*=0.05 whereas the upper sold red line indicates the significance threshold at *p=*0.01. Traits shown are **(a)** plant height in cm at 567 days, **(b)** plant height in cm at 763 days, **(c)** stem diameter in cm at 567 days and **(d)** stem diameter at 763 days.

**Additional File 5 – Figure S2 continued**

**
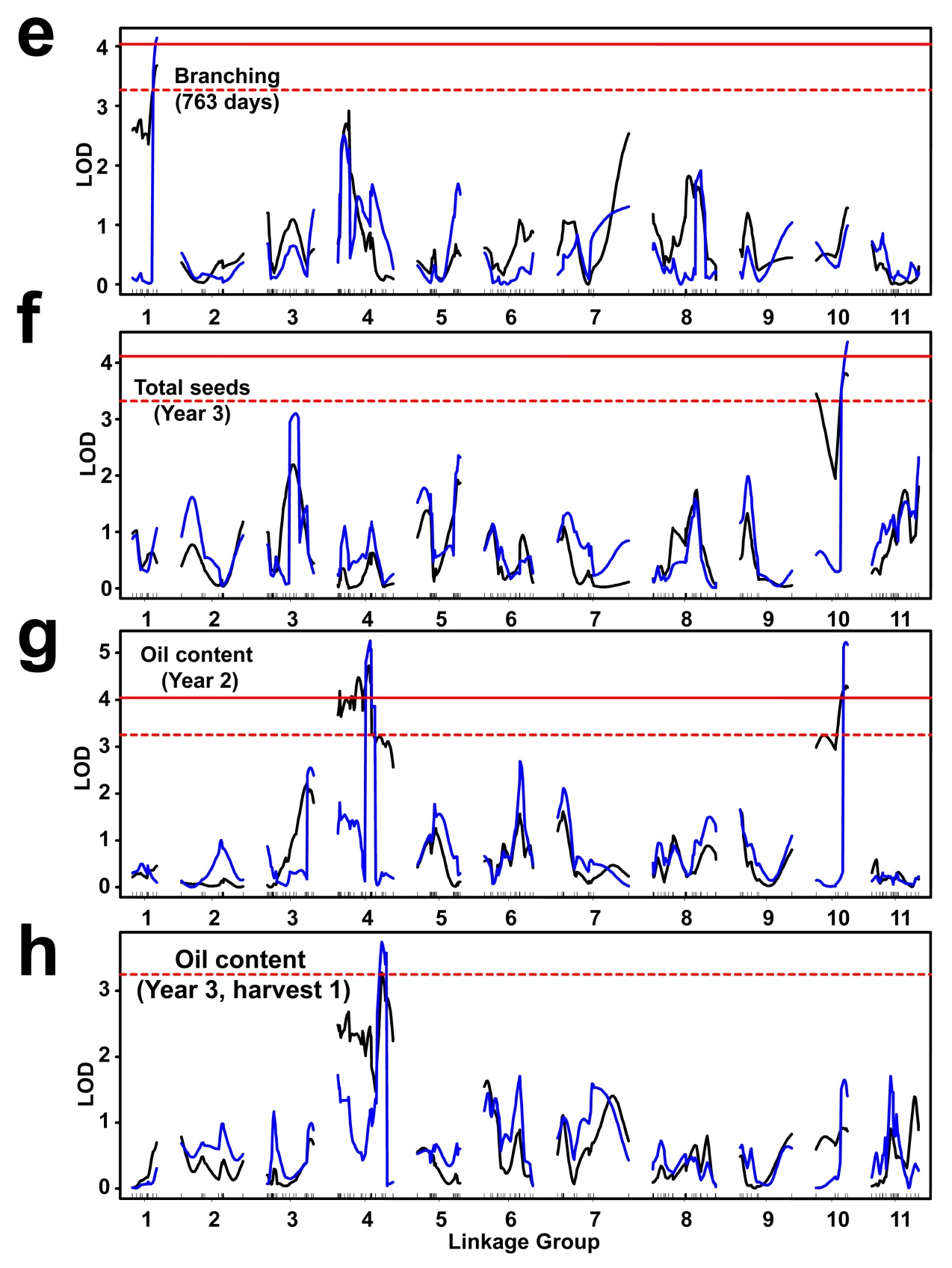
**

**Figure S2:** Outputs of initial QTL scans obtained using the Harley-Knott (black lines) and composite interval mapping (blue lines) in R/qtl. The lower dashed red line indicates the significance threshold at *p*=0.05 whereas the upper sold red line indicates the significance threshold at *p=*0.01. Traits shown are **(e)** branches per plant at 763 days, **(f)** seeds per plant in third year, **(g)** % oil content of seeds in second year and **(h)** % oil content of seeds in first harvest of third year.

**Additional File 5 – Figure S2 continued**


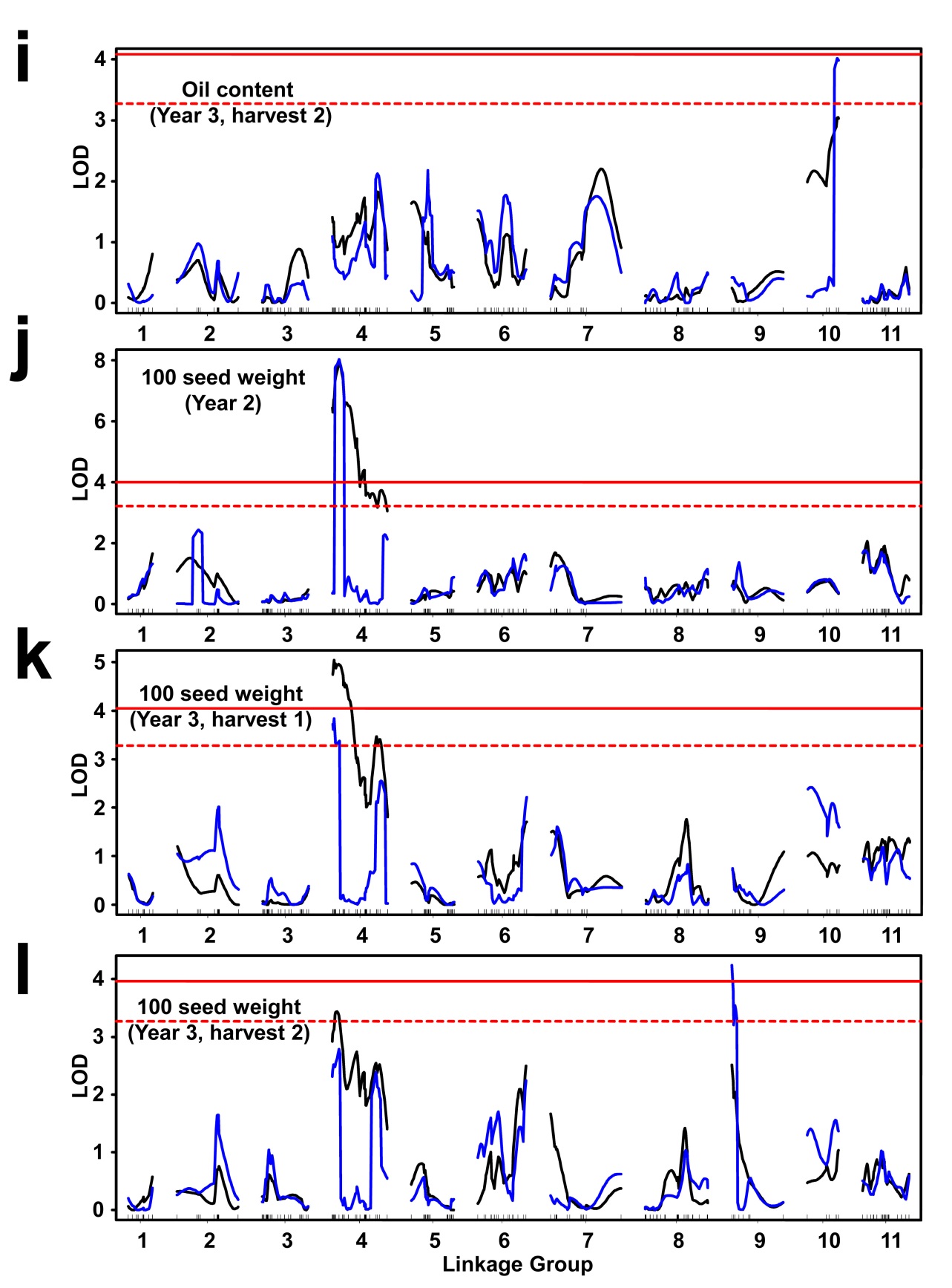


**Figure S2:** Outputs of initial QTL scans obtained using the Harley-Knott (black lines) and composite interval mapping (blue lines) in R/qtl. The lower dashed red line indicates the significance threshold at *p*=0.05 whereas the upper sold red line indicates the significance threshold at *p=*0.01. Traits shown are **(i)** % oil content of seeds in second harvest of third year, **(j)** 100 seed weight in second year, **(k)** 100 seed weight in first harvest of third year and **(l)** 100 seed weight in in second harvest of third year.

**Additional File 5 – Figure S2 continued**


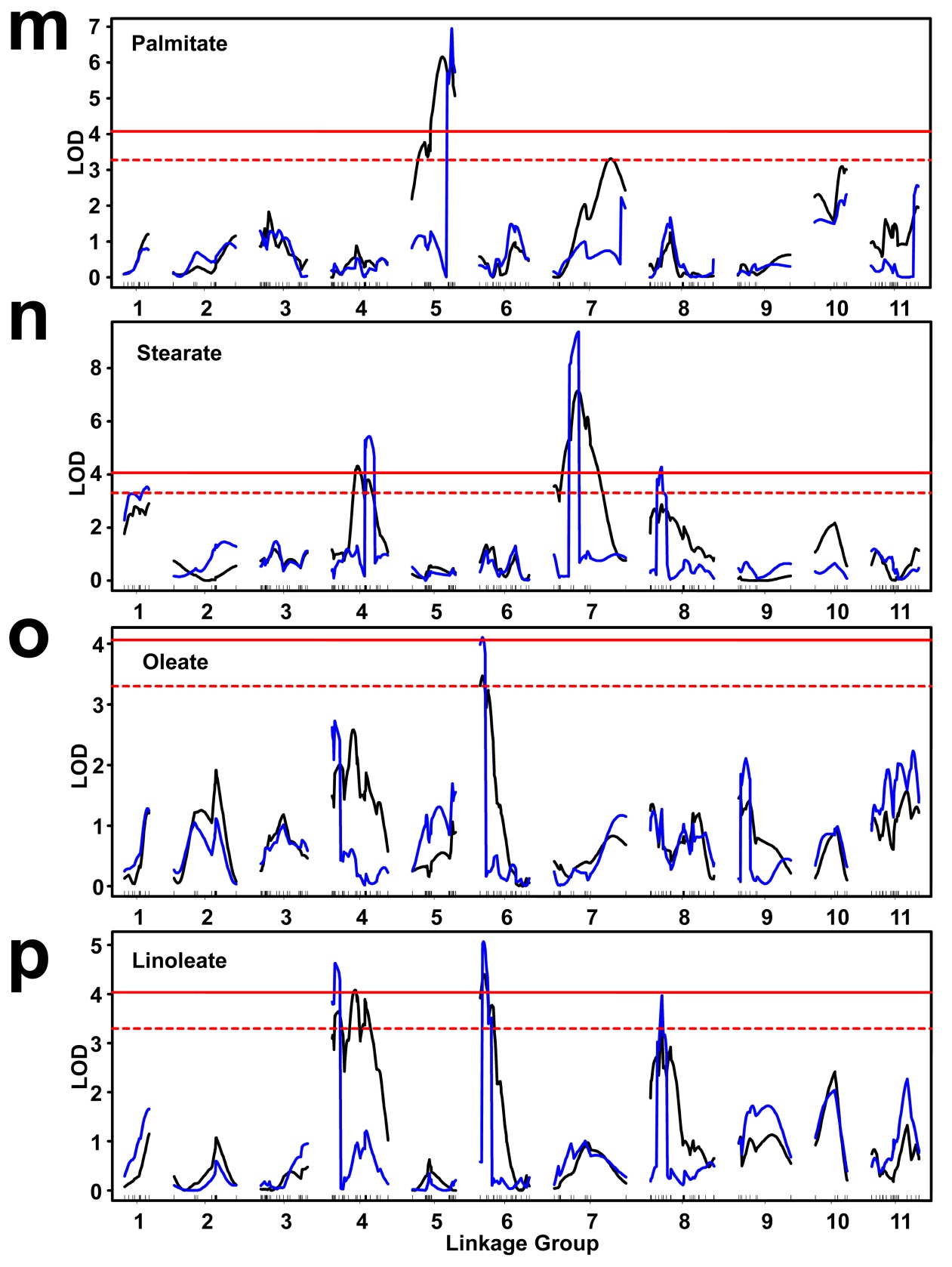


**Figure S2:** Outputs of initial QTL scans obtained using the Harley-Knott (black lines) and composite interval mapping (blue lines) in R/qtl. The lower dashed red line indicates the significance threshold at *p*=0.05 whereas the upper sold red line indicates the significance threshold at *p=*0.01. Traits shown are **(m)** % palmitate content in the seed oil, **(n)** % stearate content in the seed oil, **(o)** % oleate content in the seed oil and **(p)** % linoleate content in the seed oil.
