# Supplementary material for: Identification of QTL markers contributing to plant growth, oil yield and fatty acid composition in the oilseed crop Jatropha curcas L
Source: Biotechnol Biofuels. 2015 Sep 25;8:160. doi: 10.1186/s13068-015-0326-8 (PMC4583170; doi:10.1186/s13068-015-0326-8)
Supplement: Supplementary file 6 — Additional file 6: Figure S3. Boxplots of genotype versus phenotype for QTLs in mapping population G51 × CV [file 13068_2015_326_MOESM6_ESM.docx]

**Additional File 6: Figure S3 – Boxplots of genotype versus phenotype for QTLs in mapping population G51 x CV**

**Figure S3:** Boxplots showing distribution of phenotype against genotype data in mapping population G51 x CV at different QTL positions. The outer whiskers show the entire data range, except for outliers, which are denoted by circles. The box regions indicate the upper and lower quartiles. The central lines denote the median. The red letters indicate the results for the comparison between genotypes using Tukey’s honest significance difference test. Different letters (e.g. A and B) indicate groups found to be different a *p*=0.05 whereas an asterisk (e.g. A and A^*^) indicates that groups were found to be different at *p*=0.10. The boxplots relate to the QTL for **(a)** plant height at on linkage group 4 **(b)** and (**c)** the plant height at 763 days on linkage groups 4 and 8, **(d)** and **(e)** stem diameter at 567 days on linkage groups 4 and 5, **(f)** and **(g)** stem diameter at 763 days on linkage groups 7 and 4, **(h)** number of branches at 763 days on linkage group 1 and **(i)** seeds per plant in year 3 on linkage group 10.

**Additional File 6: Figure S3 continued – Boxplots of genotype versus phenotype for QTLs in mapping population G51 x CV**

**Figure S3:** Boxplots showing distribution of phenotype against genotype data in mapping population G51 x CV at different QTL positions. The outer whiskers show the entire data range, except for outliers, which are denoted by circles. The box regions indicate the upper and lower quartiles. The central lines denote the median. The red letters indicate the results for the comparison between genotypes using Tukey’s honest significance difference test. Different letters (e.g. A and B) indicate groups found to be different a *p*=0.05 whereas an asterisk (e.g. A and A^*^) indicates that groups were found to be different at *p*=0.10. The boxplots relate to the QTL for **(j)** and **(k)** seed oil content in year 2 on linkage groups 5 and 10, **(l)** seed oil content in year 3, harvest 1 on linkage group 4, **(m)** seed oil content in year 3, harvest 2 on linkage group 10, **(n)** 100 seed weight in year 2 on linkage group 4 **(o)** 100 seed weight in year 3, harvest 1 on linkage group 4 **(p)** 100 seed weight in year 3, harvest 2 on linkage group 4 and **(q)** and **(r)** palmitate content of seed oil on linkage groups 5 and 7.

**Additional File 6: Figure S3 – Boxplots of genotype versus phenotype for QTLs in mapping population G51 x CV**

**Figure S3:** Boxplots showing distribution of phenotype against genotype data in mapping population G51 x CV at different QTL positions. The outer whiskers show the entire data range, except for outliers, which are denoted by circles. The box regions indicate the upper and lower quartiles. The central lines denote the median. The red letters indicate the results for the comparison between genotypes using Tukey’s honest significance difference test. Different letters (e.g. A and B) indicate groups found to be different a *p*=0.05 whereas an asterisk (e.g. A and A^*^) indicates that groups were found to be different at *p*=0.10. The boxplots relate to the QTL for **(s)** palmitate content of seed oil on linkage group 10, **(t)**, **(u)**, **(v)** and **(w)** stearate content of seed oil on linkage groups 7, 4, 8 and 1 **(x)** oleate content of seed oil on linkage group 6 and **(y)**, **(z)** and (**aa)** linoleate content of seed oil on linkage groups 6, 4 and 10.
