# Supplementary material for: Identification of QTL markers contributing to plant growth, oil yield and fatty acid composition in the oilseed crop Jatropha curcas L
Source: Biotechnol Biofuels. 2015 Sep 25;8:160. doi: 10.1186/s13068-015-0326-8 (PMC4583170; doi:10.1186/s13068-015-0326-8)
Supplement: Supplementary file 7 — Additional file 7: Figure S4. Distribution of trait data recorded for mapping population G33 × G43 [file 13068_2015_326_MOESM7_ESM.docx]

**Additional File 7: Figure S4 – Distribution of trait data in mapping population G33 x G43**

**Figure S6:** Distribution of phenotypic traits in mapping population G33 x G43 including **(a)** seeds per plant in year 1, **(b)** 100 seed weight per plant in year 1, **(c)** oil content of seeds in year 1, **(d)** seed yield per plant in year 1 and **(e)** oil yield per plant in year 1.

**Additional File 7: Figure S4 continued – Distribution of trait data in mapping population G33 x G43**

**Figure S6:** Distribution of phenotypic traits in mapping population G33 x G43 including **(f)** seeds per plant in year 2, **(g)** 100 seed weight per plant in year 2, **(h)** oil content of seeds in year 2, **(i)** seed yield per plant in year 2 and **(j)** oil yield per plant in year 2.

**Additional File 7: Figure S4 continued – Distribution of trait data in mapping population G33 x G43**

**Figure S6:** Distribution of phenotypic traits in mapping population G33 x G43 including **(k)** seeds per plant in year 3, **(l)** 100 seed weight per plant in year 3, **(m)** oil content of seeds in year 3, **(n)** seed yield per plant in year 3 and **(o)** oil yield per plant in year 3.
