# Supplementary material for: Identification of QTL markers contributing to plant growth, oil yield and fatty acid composition in the oilseed crop Jatropha curcas L
Source: Biotechnol Biofuels. 2015 Sep 25;8:160. doi: 10.1186/s13068-015-0326-8 (PMC4583170; doi:10.1186/s13068-015-0326-8)
Supplement: Supplementary file 8 — Additional file 8: Figure S5. Initial QTL scans produced for traits recorded for mapping population G33 × G43 [file 13068_2015_326_MOESM8_ESM.docx]

**Additional File 8 : Figure S5 - Initial QTL scans produced for traits recorded for mapping population G33 × G43**

**Figure S5:** Outputs of initial QTL scans obtained using the Harley-Knott (black lines) and composite interval mapping (blue lines) in R/qtl. The lower dashed red line indicates the significance threshold at *p*=0.05 whereas the upper sold red line indicates the significance threshold at *p=*0.01. Traits shown are **(a)** total seeds per plant harvested in year 2 and **(b)** total seeds per plant harvested in year 3.

**Additional File 8 – Figure S5 continued**

**Figure S5:** Outputs of initial QTL scans obtained using the Harley-Knott (black lines) and composite interval mapping (blue lines) in R/qtl. The lower dashed red line indicates the significance threshold at *p*=0.05 whereas the upper sold red line indicates the significance threshold at *p=*0.01. Traits shown are **(c)** 100 seed weight in year 1, **(d)** 100 seed weight in year 2 and **(e)** 100 seed weight in year 3.

.

**Additional File 8 – Figure S5 continued**

**Figure S5:** Outputs of initial QTL scans obtained using the Harley-Knott (black lines) and composite interval mapping (blue lines) in R/qtl. The lower dashed red line indicates the significance threshold at *p*=0.05 whereas the upper sold red line indicates the significance threshold at *p=*0.01. Traits shown are **(f)** seed oil content in year 2 and **(g)** seed oil content in year 3.
