# Supplementary material for: Identification of QTL markers contributing to plant growth, oil yield and fatty acid composition in the oilseed crop Jatropha curcas L
Source: Biotechnol Biofuels. 2015 Sep 25;8:160. doi: 10.1186/s13068-015-0326-8 (PMC4583170; doi:10.1186/s13068-015-0326-8)
Supplement: Supplementary file 9 — Additional file 9: Figure S6. Boxplots of genotype versus phenotype for QTLs in mapping population G33 x G43 [file 13068_2015_326_MOESM9_ESM.docx]

**Additional File 9: Figure S6 – Boxplots of genotype versus phenotype for QTLs in mapping population G33 x G43**

**Figure S6:** Boxplots showing distribution of phenotype against genotype data in mapping population G33 x G43 at different QTL positions. The outer whiskers show the entire data range, except for outliers, which are denoted by circles. The box regions indicate the upper and lower quartiles. The central lines denote the median. The red letters indicate the results for the comparison between genotypes using Tukey’s honest significance difference test. Different letters (e.g. A and B) indicate groups found to be different a *p*=0.05 whereas an asterisk (e.g. A and A^*^) indicates that groups were found to be different at *p*=0.10. The boxplots relate to the QTL for **(a)** total seeds per plant for year 2 on linkage group 5, **(b)** and (**c)** total seeds per plant in year 3 on linkage groups 7 and 4, **(d)**, **(e)** and **(f)** 100 seed weight in year 1 on linkage groups 4, 2 and 11 and **(g)**, **(h)** and **(i)** 100 seed weight in year 2 on linkage groups 4, 11 and 2.

**Additional File 9: Figure S6 continued – Boxplots of genotype versus phenotype for QTLs in mapping population G33 x G43**

**Figure S6:** Boxplots showing distribution of phenotype against genotype data in mapping population G33 x G43 at different QTL positions. The outer whiskers show the entire data range, except for outliers, which are denoted by circles. The box regions indicate the upper and lower quartiles. The central lines denote the median. The red letters indicate the results for the comparison between genotypes using Tukey’s honest significance difference test. Different letters (e.g. A and B) indicate groups found to be different a *p*=0.05 whereas an asterisk (e.g. A and A^*^) indicates that groups were found to be different at *p*=0.10. The boxplots relate to the QTL for **(j)** 100 seed weight in year 2 on linkage group 10, **(k)**, **(l)**, **(m)**, **(n)**, **(o)** and **(p)** 100 seed weight in year 3 on linkage groups 4, 9, 11 (upper arm), 2, 11 and 10, and **(q)** and **(r)** seed oil content in year 2 on linkage groups 6 and 10.

**Additional File 9: Figure S6 continued – Boxplots of genotype versus phenotype for QTLs in mapping population G33 x G43**

**Figure S6:** Boxplots showing distribution of phenotype against genotype data in mapping population G33 x G43 at different QTL positions. The outer whiskers show the entire data range, except for outliers, which are denoted by circles. The box regions indicate the upper and lower quartiles. The central lines denote the median. The red letters indicate the results for the comparison between genotypes using Tukey’s honest significance difference test. Different letters (e.g. A and B) indicate groups found to be different a *p*=0.05 whereas an asterisk (e.g. A and A^*^) indicates that groups were found to be different at *p*=0.10. The boxplots relate to the QTL for **(s)** and (**t**) seed oil content in year 2 on linkage groups 4 and 5, and **(u)** and **(v)** seed oil content in year 3 on linkage groups 5 and 6.
